# Continuous Non-Invasive Blood Pressure Measurement Using 60 GHz-Radar—A Feasibility Study

**DOI:** 10.3390/s23084111

**Published:** 2023-04-19

**Authors:** Nastassia Vysotskaya, Christoph Will, Lorenzo Servadei, Noah Maul, Christian Mandl, Merlin Nau, Jens Harnisch, Andreas Maier

**Affiliations:** 1 Infineon Technologies AG, Am Campeon 1-15, 85579 Neubiberg, Germany; 2 Department for Computer Science 5 (Pattern Recognition), Friedrich-Alexander-University Erlangen-Nuremberg (FAU), Martensstrasse 3, 91058 Erlangen, Germany; 3 Department of Electrical and Computer Engineering, Technical University of Munich, Arcisstrasse 21, 80333 Munich, Germany

**Keywords:** FMCW radar, vital sensing, continuous blood pressure monitoring, signal processing, wearable device

## Abstract

Blood pressure monitoring is of paramount importance in the assessment of a human’s cardiovascular health. The state-of-the-art method remains the usage of an upper-arm cuff sphygmomanometer. However, this device suffers from severe limitations—it only provides a static blood pressure value pair, is incapable of capturing blood pressure variations over time, is inaccurate, and causes discomfort upon use. This work presents a radar-based approach that utilizes the movement of the skin due to artery pulsation to extract pressure waves. From those waves, a set of 21 features was collected and used—together with the calibration parameters of age, gender, height, and weight—as input for a neural network-based regression model. After collecting data from 55 subjects from radar and a blood pressure reference device, we trained 126 networks to analyze the developed approach’s predictive power. As a result, a very shallow network with just two hidden layers produced a systolic error of 9.2±8.3 mmHg (mean error ± standard deviation) and a diastolic error of 7.7±5.7 mmHg. While the trained model did not reach the requirements of the AAMI and BHS blood pressure measuring standards, optimizing network performance was not the goal of the proposed work. Still, the approach has displayed great potential in capturing blood pressure variation with the proposed features. The presented approach therefore shows great potential to be incorporated into wearable devices for continuous blood pressure monitoring for home use or screening applications, after improving this approach even further.

## 1. Introduction

High blood pressure (BP)—also referred to as hypertension—is known to be a silent killer. Considered one of the most critical risk factors for cardiovascular diseases [1,2], it can cause severe damage to human organs, mainly to the heart and kidneys, if left untreated [2,3]. Neglecting its treatment can lead to stroke, disability and, in severe cases, even premature death [4].

The gold standard for measuring BP remains the conventional upper arm-cuff device, using either the manual auscultatory or automated oscillometric method [5]. The arm cuff is inflated for both methods until blood flow is completely occluded.

For the auscultatory method, the pressure is slowly released from the cuff, and a physician will listen for so-called Korotkoff sounds while the oscillometric devices use algorithms for this purpose.

The applied cuff pressure at which the first Korotkoff sound appears is the systolic blood pressure (SBP), and the pressure at which the last Korotkoff sound appears is the diastolic blood pressure (DBP). Hence, the SBP and the DBP form the upper and lower bounds, respectively, within which the pressure wave travels during one cardiac cycle [6].

Hypertension is usually diagnosed by a physician. However, when BP is measured by a physician, it might become uncharacteristically high due to a psychological effect called Whitecoat Hypertension [5,7], referring to the white coat that physicians commonly wear. This effect, as well as the fact that automated oscillometric arm cuffs estimate rather than measure pressure [5] provide strong evidence that single measurements lack representation of the total cardiovascular state. As a direct consequence, BP must be monitored more regularly. It has been shown that daily measurements [2] or capturing BP variations over multiple measurements during one day [4] are more predictive of cardiovascular diseases. In fact, BP is one of the most dynamic physiologic variables, as pulse waves continuously change in terms of both frequency and amplitude [5]. A myriad of external factors influences BP—food intake, stress, age, weight, body height, medication, and body position, to name a few [8]. Hence, it not only varies over long periods of time but also fluctuates over short time frames.

### 1.1. Blood Pressure Physiology

**Figure 1:**
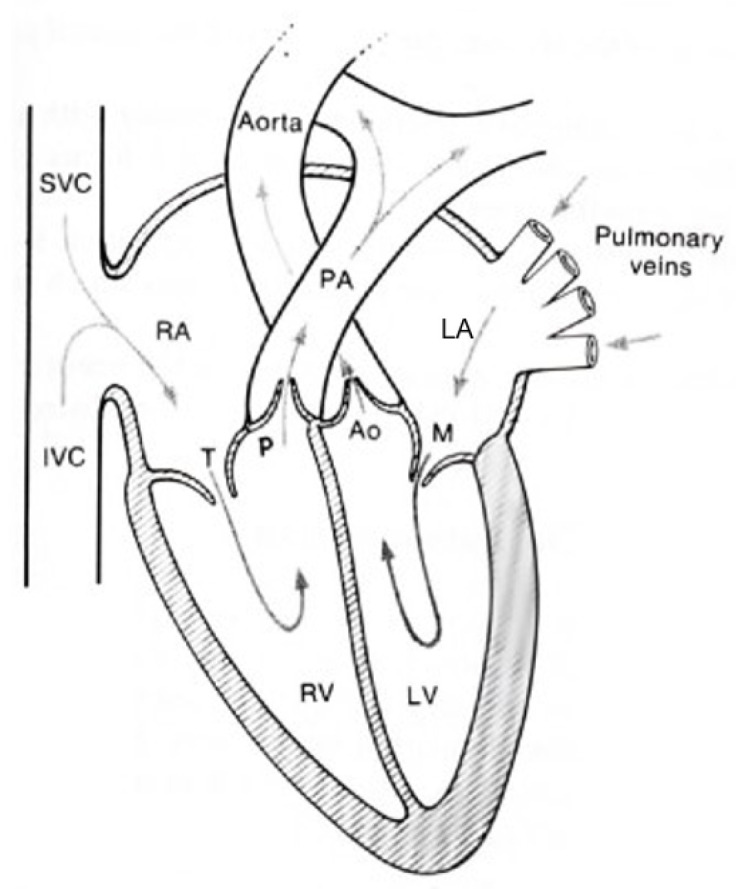
Blood flow circulation through heart chambers, valves, arteries, and veins. Adapted with permission from [9]. *©* 1990, Springer Science+Business Media New York.

The heart is a periodic fluid pump [5]. It pumps blood to transport nutrients and oxygen through the body. The blood circulation is depicted in Figure 1.

Blood that needs oxygenation is pumped to the lungs through the pulmonary artery (PA), where it is oxygenated and then returns to the heart via the pulmonary veins [9]. It then passes the left atrium (LA) through the mitral valve (M) into the left ventricle (LV), from where it is pushed into the aorta—a large artery from which the arterial tree spans—through the aortic valve (AO) [9]. This oxygenated, nutrient-rich blood travels through this arterial tree.

It then returns to the heart via the superior vena cava (SVC) and inferior vena cava (IVC) veins back to the heart into the right atrium (RA) [9]. In the RA, the blood is pushed to the right ventricle (RV) through the tricuspid valve (T), and the cycle begins anew [9].

When the blood is pushed through the arterial tree, it exerts force onto the arterial walls—this is the reason it is called BP. In order to push blood into the aorta, the heart needs to build up pressure in the LV, where the highest pressure marks the systolic pressure, right at the opening of the aortic valve [9,10]. Consequently, the lowest pressure—the diastolic pressure—occurs when the aortic valve closes and blood flow is stopped [5].

**Figure 2:**
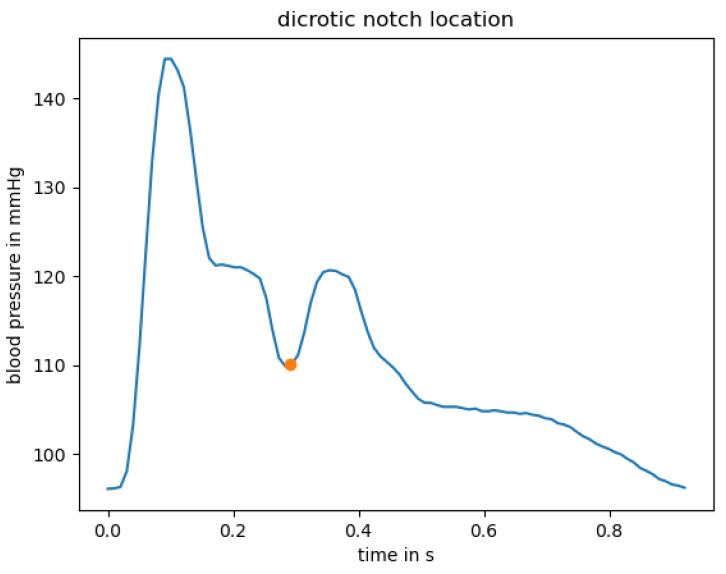
Example pressure pulse wave from the radial artery. Dicrotic notch location is marked with orange dot.

The BP curve, however, is not linear from systolic to diastolic pressure and back: It rather constitutes “a series of harmonics travelling along the arterial tree, rather as on a plucked violin string” [10]. A typical waveform taken from the radial artery can be seen in Figure 2. The beginning of the curve—the lowest pressure—marks the diastolic pressure.

When the heart pushes blood into the arterial tree, this leads to a steep increase in pressure up to the systolic pressure. After that, the pressure begins to decrease non-linearly. The pressure waveform is, in fact, a superposition of the pressure wave generated from the LV and a reflection of the wave at peripheral sites back to the aorta, resulting in an augmentation of pressure [10]. This augmentation leads to a trough before a second upstroke in pressure due to this reflection, which is called dicrotic notch [5].

Figure 2 shows an example location of the dicrotic notch at around 300 ms, visualized with an orange dot.

BP is historically measured in millimeters of mercury, short mmHg. The typical, healthy BP value is 120 mmHg systolic over 80 mmHg diastolic pressure [1]. People with elevated BP should monitor their BP regularly as they are likely to develop hypertension [11]. Methods for reducing BP include regular exercise, a balanced diet or medication. If systolic BP values exceed 180 mmHg, a doctor should be contacted immediately [11].

### 1.2. Contributions

To the best of the authors’ knowledge, this is the first publication of its kind to provide a full tutorial-style signal processing pipeline for continuous radar-based blood pressure estimation. The work covers extracted features for pulse wave analysis-based neural network regression and feature justification in detail.

Additionally, this work proposes a beamforming algorithm used for improving signal-to-noise ratio. This research was conducted as a potential commercial application, as it shows that even a small network topology produces meaningful results, hence allowing implementation on an embedded microcontroller. While we did not reach clinical accuracy, the proposed approach shows great potential and could be employed in a wearable device for at-home usage, allowing seamless integration into daily life.

### 1.3. Article Structure

The rest of the journal article is structured as follows: Section 2 presents analysis-based work and the motivation for the need for a novel blood pressure monitoring system. Section 3 gives a detailed introduction to radar fundamentals. Section 4 describes signal processing techniques to extract pulse waves from the underlying skin displacement, Section 5 covers data collection, feature extraction, and training of various neural network topologies. Section 6 describes the blood pressure mapping results, which are then discussed in Section 7. The work is concluded in Section 8.

## 2. Related Work

A myriad of blood pressure monitoring approaches exist. Nevertheless, no ideal solution has been established [5]. The ideal blood pressure monitoring technique should be non-invasive, accurate, relatively inexpensive, and use a sensor small enough to be integrated into wearable devices to allow for daily readings. Additionally, it should not cause discomfort, should allow and provide continuous BP readings and extract the whole pulse waveform, not just upper and lower pressure bounds, since the waveform contains a multitude of information about the patient’s cardiovascular state. Given the importance of such devices for medical diagnosis and preventative care, optimizing BP measuring technology is an active area of research. A few examples are given in the following.

Applanation tonometry can be used to extract individual pulse waves due to its inherent pressure-sensing nature [12]. This technology provides continuous readings while being non-invasive. Therefore, it gives both morphological information and directly returns BP readings for each pulse wave. However, this technology suffers from severe limitations: Applying a tonometer pen inflicts discomfort and pain. Additionally, a trained physician needs to hold the tonometer in perfect position. This dramatically restrains everyday life. Thus, this approach seems only feasible in a clinical setting for a short period of time.

Photoplethysmography (PPG) is an optical technology that is already being used in pulse oximeters [5]. It measures the pulsatile change of blood volume [5,13] by measuring the light intensity return of the light that is emitted through the tissue using an LED [5,14]. While the PPG cannot measure pressure itself [2], research has proven that the PPG waveform reflects the radial pressure waveform and shows similar morphological changes in diseases [5,13].

In [6], the authors operated on the open-source clinical MIMIC database, which contains synchronized electrocardiograph (ECG) signals, arterial blood pressure waveforms, fingertip PPG signals, and reference SBP and DBP, among others [15]. The authors claimed that the amplitude of the PPG signal varies significantly and therefore excluded amplitude-based features for their approach. Instead, they focused on timing-based features and extracted 21 timing differences and timing ratios as input for a neural network. Their results are very promising, with errors of 3.8±3.46 mmHg and 2.21±2.09 mmHg (mean error ± standard deviation) for SBP and DBP, respectively. PPG technology is “non-invasive, non-occlusive, low-cost, and already in use clinically as part of standard monitoring equipment” [16], making it a good option for extracting pulse waveforms for BP estimation. However, the PPG is influenced by environmental factors such as temperature [13] or skin pigmentation. In fact, the concentration of melanin in human skin can attenuate the incident light, as melanin is highly light absorbent [17].

A larger melanin concentration will therefore lead to smaller pulse heights [17].

The study in [17] has shown that Fitzpatrick skin type group V (dark brown) demonstrated significantly lower modulation than other groups. This difference in pulse heights would consequently change amplitude features of extracted pulse waves—as those features are used to estimate blood pressure values, this could likely falsify those estimates.

Due to this—unintended, however present—discrimination of people of color, causing lack of measurement accuracy and reliability, this technology is insufficient for inclusive patient monitoring.

In [18], the authors used imaging photoplethysmography (iPPG) techniques to track subtle changes in skin color, corresponding to blood pressure curves. For each curve they extracted temporal, and amplitude features, areas, and derivatives and compared the locations of these imaging PPG features to those of contact PPG ones, after further processing the iPPG pulse waves. This technique shows promising correlation of the imaging based wave extraction and the contact based one. However, no results for mapping to blood pressure are presented in their work. A limitation of their presented work is that the forehead area must be well-illuminated and the subject must sit still in front of the camera. Thus, it is not seamlessly integratable into daily life.

In [19], the authors created a two-channel ballistocardiogram (BCG) by placing polyvinylidene fluoride resin film inside a sofa so that BCG signals from the subject’s back and thighs are captured. They extracted the instantaneous phase of the signals by applying a Hilbert Transform on the first intrinsic mode function of the BCG signals that was obtained using empirical mode decomposition. They used a 1D-CNN architecture to train BP from this instantaneous phase. In resting position, they achieved an error of 0.93±6.24 mmHg for systole and 0.21±5.42 for diastole. While these are great results, this approach suffers from the following limitations: Only non-hypertensive subjects were selected for the study—this limits the expected blood pressure range significantly and thus makes prediction much easier. Additionally, they only measured the cuff-based reference BP five times per minute in a resting state, thereby drastically limiting the data amount for training and failing to capture beat-to-beat variations.

Lastly, radar technology was also increasingly discussed in the literature.

In [20], a 900 MHz continuous wave (CW) radar was placed on the sternum to extract displacement information from the aorta. This displacement contains the pulse waves. The authors used a technique called pulse transit time (PTT) for BP estimation. For that, they used the timing difference between the R-peak of the electrocardiograph (ECG) and the foot of the radar-based pulse waves as an estimate for the pre-ejection period (PEP) which is essentially the time required to build pressure for the blood to be pushed into the body. They extracted the pulse arrival time (PAT) as the timing difference between the R-peak of the ECG and the maximum slope of a PPG attached to the earlobe. Then, they calculated the PTT by subtracting the PEP from the PAT and used a linear model to estimate the BP using the PTT. While this result demonstrated reasonably accurate results, with the cumulative error percentage (in this case, the percentage of predictions where the error was below a given threshold) being below 7 mmHg for 81.3% and below 15 mmHg for 92.28%, this approach lacks simplicity. Three different sensor technologies are employed, rendering this solution bulky and expensive. Additionally, ECG lacks convenience for personal use as it is best applied by a physician.

The authors of [21] utilized a 60 GHz frequency-modulated continuous wave (FMCW) radar in a wristwatch-like enclosure with a standoff distance of 1 cm. While they demonstrated that the radar system can extract the pulse waveform and some of its important morphological features, they did not use the extracted pulse waveforms to extract features and use those to map the curves to blood pressure values.

In [22], a 24 GHz continuous wave radar was used to extract pulse waves at 15 cm from the chest. After that, six features are extracted per pulse wave—three are timing features, two are amplitude related, and the sixth is the heart rate. While this method produced excellent results with errors of 0.22±3.85 mmHg and 2.52±6.73 mmHg for DBP and SBP, respectively, it suffers from limited integration in daily life: Patients needed to sit completely still on a chair, 15 cm away from the radar. Therefore, such a setup could not be used to monitor trends in BP variation during daytime activities or during sleep.

Hence, it can be concluded that no ideal solution exists to date. Referring back to the beginning of this section, we chose a 60 GHz FMCW radar to evaluate whether it satisfies all criteria for an ideal blood pressure monitoring solution. As the chosen sensor is non-invasive, small, and inexpensive, the aim was to show whether the use of radar technology allows the extraction of pulse waveforms and their mapping to BP values accurately. In the following, we provide a digital signal processing tutorial for blood pressure curve extraction along with a signal-to-noise ratio improvement procedure. We propose a set of features to be extracted from an individual pulse wave and show that these contain enough meaningful information that using a very small neural network shows good predictive power.

## 3. Radar Fundamentals

The ideal BP technique should be non-invasive, accurate, inexpensive, comfortable, small, provide continuous BP readings, and extract the whole pulse waveform.

The radar inherently meets some criteria. It is an inexpensive technology that requires a low power supply and can be very small. When integrated into a wearable device, it becomes invisible—no emitted light, no camera lenses.

Therefore, it complies with data privacy protection because subjects cannot be distinguished with only the radar’s raw data. Additionally, the radar is independent of environmental factors such as ambient temperature, light source, weather, or the skin’s melanin concentration.

Radar is an acronym for radio detection and ranging [23]. A radar generates electromagnetic waves and transmits those into its surroundings via the transmit antennas. When those waves hit a target, they are partially reflected and then detected by the receivers.

Continuous wave (CW) radars can be used to extract information about a target’s movement and its radial velocity, meaning its velocity relative to the radar. If a target moves towards the radar, the reflected waves superposition each other and produce a reflected wave of higher frequency than the transmitted wave. Similarly, if the target moves away from the radar, the reflected wave has a lower frequency than the transmitted one [24]. A practical example is an ambulance—the more it approaches, the more high-pitched the sound becomes due to the superposition and thus increase of sound frequencies. This is a well-known phenomenon called the Doppler Effect.

Movement can therefore be extracted by using this Doppler Effect—if the returned frequency differs from the transmitted one, the target exhibited movement. This shift in frequency, the Doppler shift, can be used to extract the radial velocity of this moving target by using the relation

(1) $$f_{D}=\frac{2\cdot f_{\mathrm{radar}}\cdot v\cdot \cos\left(\theta \right)}{c}$$

for velocity estimation [25]

(2) $$v=-\frac{\lambda \cdot f_{D}}{2}.$$

Compared to a CW radar, a frequency-modulated CW (FMCW) radar has the additional advantage of having range resolution due to its changing emitted frequency [21].

We thus chose an FMCW radar due to the close proximity of the radar to the skin when embedding the sensor into a wearable device—this way, only the closest-range return signal can be used for further signal processing. As the name suggests, the transmitted frequencies vary over time—the most common frequency pattern is the saw-tooth pattern with a linear increase from lower to upper frequency and an abrupt decrease back to the lower frequency. The difference between the upper and lower frequencies is called the bandwidth *b*,

(3) $$b=f_{\mathrm{upper}}-f_{\mathrm{lower}}=\left(f_{0}+b\right)-f_{0}.$$

**Figure 3:**
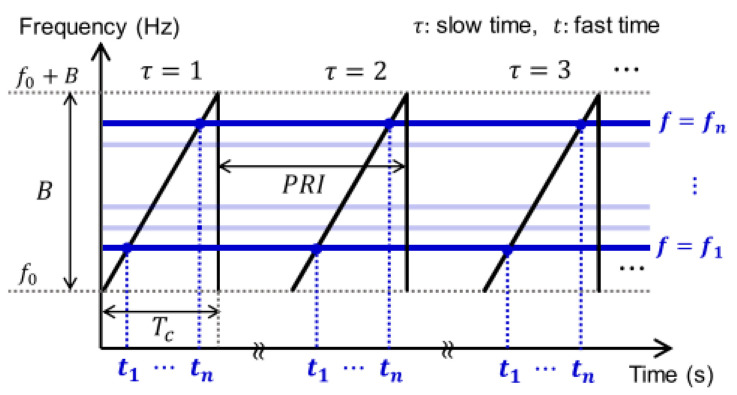
Example of transmitted chirp-signal frequency pattern from FMCW radar. Reprinted with permission from [26]. *©* 2020, IEEE.

An illustration of this functionality is given in Figure 3—the frequency is linearly increasing over time, starting at the lower frequency $f_{0}$ at the fast sampling time step $t_{0}$ and iteratively increasing its frequency with each sampling time step $t_{i}$, with i∈[0,n−1], where *n* is the number of samples per chirp, until the upper frequency is reached. An increase in frequency is equal to a faster oscillation of the transmitted signal.

The choice of upper and lower frequencies will affect the wavelength of the electromagnetic waves. The higher the emitted frequencies, the smaller the wavelength and the more sensitive the radar is to motion.

The wavelength can be computed as

(4) $$\lambda =\frac{c}{f_{c}},$$

where *c* is the speed of light, and $f_{c}$ is the center frequency

(5) $$f_{c}=f_{0}+\frac{b}{2}.$$

The fast time is defined by the number of samples per chirp, while the slow time refers to the number of chirps per second—the reason for this naming is the short duration of the sampling compared to the execution of the whole chirp. The pulse repetition interval PRI, also depicted in Figure 3, is the pulse repetition time (also known as PRT) and is the time distance between two consecutive chirps.

**Figure 4:**
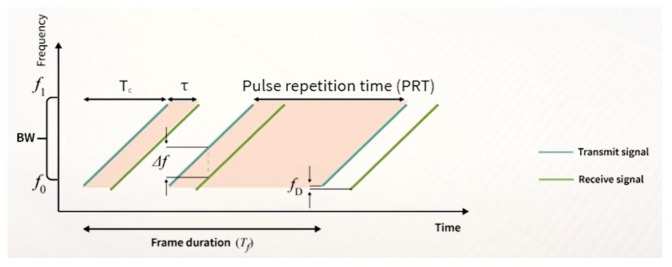
Visualization of the relation between transmitted and received waves. Source: [25].

The time it takes for the chirp to reach the upper frequency from the lower frequency is called the chirp duration $t_{c}$—this is depicted in Figure 4.

As described above, an FMCW radar has the advantage of having range resolution due to its changing emitted frequency [21]. This way, the distance of a target relative to the radar can be calculated, and the reflected signal from only a specific range bin or a span over multiple range bins can be used. This is especially useful when the radar’s range exceeds the application’s region of interest and might cause unnecessary noise.

Additionally, this allows for spatial discrimination of targets [21]. The range resolution determines the degree to which this spatial resolution is possible—it can be computed as [27]

(6) $$\Delta r=\frac{c}{2b},$$

where *c* is the speed of light, and *b* is the bandwidth. The distance of a target relative to the radar can be calculated by using the timing difference between the transmitted and received signal τ, also referred to as round trip delay, as [27]

(7) $$r=\frac{c\tau }{2}.$$

The maximum distance can be obtained by [28]

(8) $$r_{\max}=\frac{f_{s}t_{c}c}{2b},$$

where $f_{s}$ is the sampling rate, and $t_{c}$ is the chirp duration.

The transmitted FMCW signal is expressed as

(9) $$TX\left(t\right)=a_{\mathrm{TX}}\cos\left(2\pi f_{0}t+\pi \frac{b}{t_{c}}t^{2}+\theta \left(t\right)\right)$$

and the received signal from a target in range *r* as

(10) $$RX\left(t\right)=a_{\mathrm{RX}}\cos\left(2\pi f_{0}\left(t-\tau \right)+\pi \frac{b}{t_{c}}{\left(t-\tau \right)}^{2}+\theta \left(t-\tau \right)\right),$$

where $a_{\mathrm{TX}}$ and $a_{\mathrm{RX}}$ are the amplitude of the transmitted and received signal, respectively, *t* is time, τ is round trip delay depending on the distance *r* and θ is the phase noise [28].

In practice, however, we did not have access to the round trip delay. Instead, we computed the so-called range FFT (Fast Fourier Transform) to extract the beat frequency—which is visualized as Δf in Figure 4—and the Doppler shift $f_{D}$. The beat frequency $f_{B}$ is the frequency difference between the transmitted and received waves. In comparison to the beat frequency, the Doppler shift is very small.

The range FFT was performed per chirp and, thus, along the fast samples/chirp time axis, not the slow chirps/second time [26,28,29]. It yields the spectrum of the beat signal, where peaks at a target bin identify targets at that particular distance [28]. The complex output of the range FFT at those target bins contains the phase of the returned signal at that specific range bin and thus contains the same information as for a CW radar. Hence, the phase can be used to extract both motion and velocity. This property was utilized, as explained in the following section, for extracting skin displacement.

## 4. Methods

**Figure 5:**
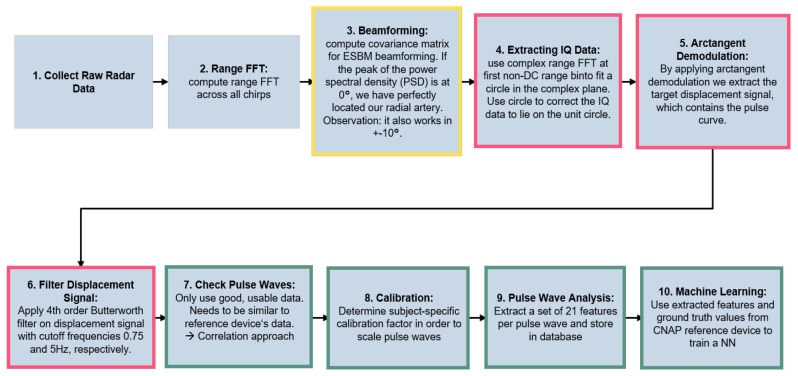
Employed signal processing pipeline. The boxes are colored according to their method subsection.

This section gives an overview of the methods employed for extracting skin displacement. Figure 5 outlines the complete pipeline of the proposed algorithm for radar-based blood pressure estimation. The pseudocode for the pipeline is given in Algorithm 1.

The output from the receiver antennas is referred to as the radar’s raw data. These raw data are then used for optimally placing the radar sensor with the help of digital beamforming (described in detail in Section 4.1) and for extracting the skin displacement (see Section 4.2). The chain of thought for our skin displacement extraction approach was the following: when blood is pumped through the artery, it expands.

Since the skin is elastic, this motion will propagate to the skin surface which we can then measure with radar. This process is called vasomotion [30,31].

Finally, Section 4.3 covers the proposed feature extraction for mapping to blood pressure values.
**Algorithm 1** Pseudocode for raw data processing1:**procedure** RawDataProcessing(radar)2:    raw_data←Get_Raw_data(radar)3:    previous_circles←[]  4:*loop*:5:    doa←ESBM(raw_data)6:    pulse_waves←DisplacementExtraction(raw_data, previous_circle)7:    features←FeatureExtraction(pulse_waves)8:    BP_values←MapFeaturesToBP(features)9:    goto *loop*.

### 4.1. Locating the Radial Artery

Before attempting to extract skin displacement, it is crucial to locate the radial artery in order to improve the signal-to-noise ratio. The phase difference between multiple receive antennas can be used to estimate the precise angle from which a signal is emitted [25]:

(11) $$\Delta \phi_{rx1,rx2}=\frac{2\pi d\cdot \sin(\theta )}{\lambda }.$$

This is known as Direction-of-Arrival (DOA) estimation.

The wavefield arrives at an m-element uniform linear receiver antenna array from an angle θ, which is the angle of the target relative to the radar. The number of different target directions *d* that can be distinguished is limited as

(12) $$d_{\max}=m-1.$$

In this work, the digital beamforming algorithm, called the Eigenspace-based Method (ESBM) [32]—an optimization of the well-known MVDR (Minimum-Variance Distortionless Response) algorithm [33]—was used to estimate the DOA. The advantages compared to conventional MVDR beamforming are discussed in [32].

The pseudocode for calculating the DOA is outlined in Algorithm 2.
**Algorithm 2** Pseudocode for ESBM-based DOA estimation1:**procedure** ESBM(**raw_data**)  2:    antenna1_response←FFT_first_bin(raw_data_from_antenna1)3:    antenna3_response←FFT_first_bin(raw_data_from_antenna3)4:    X←[antenna1_response;antenna3_response]  5:    $R\leftarrow X*X^{\prime }$6:    R_inv←Inverse(R)  7:    [eigenvectors,eigenvalues]←EVD(R)8:    [eigenvectors,eigenvalues]←Sort(eigenvectors,eigenvalues)9:    A←eigenvector_of_largest_eigenvalue  10:*loop over angle range of interest*:11:    a←Steeringvector(angle)12:    $a\_\mathrm{proj}\leftarrow A*A^{H}*a$13:    $w\_\mathrm{proj}\leftarrow \frac{R\_\mathrm{inv}*a\_\mathrm{proj}}{a^{\prime }*R\_\mathrm{inv}*a}$14:    $\mathrm{psd}\_\mathrm{for}\_\mathrm{angle}\leftarrow {w\_\mathrm{proj}}^{\prime }*R*w\_\mathrm{proj}$15:    gotoloopoveranglerangeofinterest.  16:    doa←Peak_angl(psd)  17:    returndoa

First, the covariance matrix R must be calculated by stacking the array response and multiplying it with its Hermitian as in Equation (13):

(13) $$R=E[xx^{H}],$$

where *H* signifies that the receiver signal x is multiplied by its Hermitian to obtain covariance. The array response was extracted from the target range bin’s range FFT of the raw data from antenna 1 and antenna 3, since these two antennas formed a uniform linear array. Since the response of two antennas was used, R will have the dimension 2×2.

The ESBM method projects the steering vectors into the signal subspace A in order to improve the signal-to-noise ratio and thus the DOA performance [32]. The signal subspace can be obtained from the covariance utilizing eigenvalue decomposition—the *m* eigenvalues are sorted in descending order. The eigenvectors belonging to the *d* largest eigenvalues span the signal subspace, and the other ones span the noise subspace. In this case, the signal subspace A is extracted by using the eigenvector belonging to the largest eigenvalue of R as only one target is considered.

Digital beamforming algorithms use so-called steering vectors that describe phase shifts when a planar wave hits the receiver array depending on its angle to determine the DOA of a target.

Therefore, a loop over the angle range of interest is created. In this case, angles from −40° to +40°, with 1° step size, were selected. This was considered sufficient due to the very close proximity to the skin.

Under the assumption that the distance between two antennas is $d=\frac{\lambda }{2}$, the steering vectors a are calculated as

(14) $$a\left(\theta \right)={\left[1\cdots e^{-j\pi (m-1)sin(\theta )}\right]}^{T}.$$

For each angle, its correspondent steering vector was calculated using Equation (14) and then projected into the signal subspace *A* with Equation (15) in order to improve the signal-to-noise ratio:

(15) $$a_{\mathrm{proj}}={\mathrm{AA}}^{H}a.$$

To determine the DOA, the Power Spectral Density (PSD), which gives the mean square value of wave amplitudes arriving at the sensor array, was computed:

(16) $${\mathrm{psd}}_{\mathrm{ESBM}}=w_{\mathrm{proj}}^{H}Rw_{\mathrm{proj}}.$$

The angle that maximizes this PSD is returned as the angle of arrival. Since the steering vectors were projected into the signal subspace, the beamforming weights needed to account for that. The ESBM weights adapt to their environment in such fashion that the PSD of DOA waves—with its corresponding steering vector $a_{\mathrm{proj},\mathrm{signal}}$ with the respective DOA $\theta_{\mathrm{signal}}$—remains unchanged. At the same time, the PSD of all non-signal directions is minimized [32].

This is formulated in a minimization problem of the PSD,

(17) $$\min_{w_{\mathrm{proj}}}w_{\mathrm{proj}}^{H}Rw_{\mathrm{proj}}\;s.t.\;w_{\mathrm{proj}}^{H}a_{\mathrm{proj},\mathrm{signal}}=1.$$

The optimum weights $w_{\mathrm{proj}}$ after solving this minimization problem using a Lagrange-polynomial are

(18) $$w_{\mathrm{proj}}=\frac{R^{-1}a_{\mathrm{proj}}}{a_{\mathrm{proj}}^{H}R^{-1}a_{\mathrm{proj}}}.$$

This way, the amplitudes of non-signal direction waves are suppressed, and only signal direction waves can pass unhindered. The PSD is thus a function of weights that depend on the projected steering vectors, which in turn depend on their respective angle. In conclusion, a peak of the PSD at a specific angle signifies that a signal is arriving from that specific angle.

**Figure 6:**
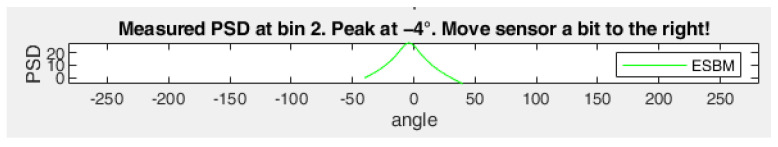
Example of returned PSD. Target detected at −4° relative to the radar.

By applying peak search over the PSD of the returned signal, the precise angle of signal arrival could be calculated. An exemplary output of the calculated PSD can be seen in Figure 6, where the target is detected at −4°.

This information was then used to shift the radar placement if needed so that the radial artery was located directly under the radar. This ensured the recording of skin displacement rather than noise. However, the area from which the signal reflects is very large due to the proximity. Therefore, in this work, a DOA range of [−10°;10°] was considered a suitable radar positioning.

The next step in the data acquisition pipeline is skin displacement extraction.

### 4.2. Skin Displacement Extraction

Radars are capable of extracting very small movement [30]. The radar continuously emits frequency waves—when these emitted waves hit a target undergoing periodic motion, the phase of the reflected waves contains this movement [4]. As described before, the phase of the beat frequency can be used to extract motion [34]. Even movements that are significantly smaller than the range resolution can be extracted [21], as the reflected wave is phase-modulated by this motion and thus contains its respective frequency components [4]. The beat frequency can be calculated as [27,28]

(19) $$f_{B}=\frac{2br}{ct_{c}}.$$

CW radars use a homodyne quadrature mixer that uses digital signal processing to yield in-phase (I(t)) and quadrature (Q(t)) baseband signals [4] that contain the phase of the signal, which includes the motion. They can be expressed as

(20) $$I\left(t\right)=a_{I}\cos\left(2\pi f_{B}nt_{m}+\psi_{l}\right)+dc_{I}$$

and

(21) $$Q\left(t\right)=a_{Q}\sin\left(2\pi f_{B}nt_{m}+\psi_{l}\right)+dc_{Q},$$

respectively [28], where $a_{I}$ and $a_{Q}$ are the I/Q channel amplitudes, $f_{B}$ is the beat frequency, *n* is the number of samples per chirp, $t_{m}$ is frame duration, $\psi_{l}$ is the phase information, and $dc_{I}$ and $dc_{Q}$ are the DC offsets.

FMCW radars do not have a quadrature mixer and therefore use the complex output of the range FFT target bin as substitutionary I(t) and Q(t) baseband signals. These signals are orthogonal to each other in order to avoid the so-called *nullpoint problem*.

If the incoming signal causes one baseband signal to vanish essentially and thus to have the least sensitivity, the other baseband has full sensitivity due to this orthogonality [35]. This way, losing a signal can be avoided.

In contrast to the DOA estimation (Section 4.1), displacement extraction only needs the FFT output from a single antenna.

Since the radar is in closest proximity to the skin, the complex output of the FFT of the first range bin was utilized as substitutionary I(t) and Q(t) signals (second row of Algorithm 3). These signals lie on a circle in complex plane, with the DC offsets $dc_{I}$ and $dc_{Q}$ being the center coordinates and the I/Q channel amplitudes $a_{I}$ and $a_{Q}$ determining the radius. However, since the skin displacement is much smaller than the radar wavelength, the I/Q data will not form an entire circle but only an arc [29,36] to which the circle must be fitted.
**Algorithm 3** Pseudocode for displacement extraction1:**procedure** DisplacementExtraction(**raw_data**,**previous_circles**)  2:    range_FFT←FFT(raw_data_from_antenna3)3:    iq←range_FFT_first_bin4:    a,b,r←CirclefitbyTaubin(iq,previous_circle)  5:    $\mathrm{compensated}\_\mathrm{IQ}\leftarrow \mathrm{Complex}(\frac{\left(\mathrm{Real}(\mathrm{iq})-a,\;\mathrm{Imag}(\mathrm{iq})-b\right)}{r})$  6:    phase←Unwrap(ArctangentDemodulation(compensated_IQ))7:    pulsewaves_rad←ButterworthFilter(data=phase,order=4,cutoff=[0.75,5])8:    pulsewaves←pulsewaves_rad∗t  9:    returnpulsewaves

This increases the difficulty of fitting a circle. In order to apply DC offset compensation, a circle must be fitted to the substitutionary I(t) and Q(t) signals (third row of Algorithm 3). This was carried out by following the circle fit estimation by Taubin as outlined in Algorithm 4.

Generally, fitting a curve to a set of data points can be performed by minimizing the mean square distance of the curve to a set of given data points [37]. In [37], however, Taubin proved that—in the case of planar curves—this problem can be simplified by solving a generalized eigenvector problem.
**Algorithm 4** Pseudocode for circle fit estimation1:**procedure** CircleFit(**iq**, **previous_circles**)  2:    n←Length(iq)3:    centroid_I,centroid_Q←Mean(iq)4:    i←Complex(iq)−centroid_I5:    q←Imag(iq)−centroid_Q6:    $z\leftarrow i^{2}+q^{2}$  7:    M←Covariance(i,q,z)/n8:    a←coefficients_characteristic_polynomial  9:*loop*:10:    y_new=a(1)+x_new∗(a(2)+x_new(a(3)+x_new∗a(4)))11:    $x\_\mathrm{new}=x\_old-\frac{y\_\mathrm{new}}{\mathrm{Dy}}$12:    **if** (x_new−x_old)/x_new<eps **then**13:        break14:    goto *loop*.  15:    a,b,r←estimatedbyusingeigenvectorrelatedtorootx_new16:    a,b,r←[a+centroid,b+centroid,r]17:    previous_circles←runningbufferoverlast30estimatedcircles18:    a,b,r←Mean(previous_circles)  19:    returna,b,r

First, the input was centered by subtracting its mean value, stabilizing the solution [37]. Then, the covariance matrix of the I/Q input and their circle representation ($I^{2}+Q^{2}$) was estimated and normalized by the number of data samples *n*. The generalized eigenvector problem is formulated as

(22) $$f_{i}M=\lambda_{i}f_{i}N,$$

where $f_{i}$ are the eigenvectors, $\lambda_{i}$ eigenvalues, M is the covariance matrix of input data samples—referred to as the “matrix of moments” [38]—and N is the covariance matrix of the Jacobian matrix of the input data samples. The eigenvector problem was solved by finding the root of the characteristic polynomial. The algorithm is roughly outlined in Algorithm 4, for more details please see [37]. In row 10 of Algorithm 4, the characteristic polynomial was evaluated using the coefficients of the polynomial and the root estimate $x_{\mathrm{new}}$.

In order to find the root of this polynomial, the solution is iteratively updated by applying the Newton-Raphson root-finding algorithm [37]. In each iteration, the root estimate $x_{\mathrm{new}}$ was updated until the value difference between two consecutive estimates was less than some pre-defined threshold value eps, i.e., the solution has converged to the root. After that, the eigenvector related to the eigenvalue was computed and used to estimate the fitted curve center (a,b) and radius *r*. The circle center was then calculated by adding the mean of the data points back to a and b, respectively. More implementation details are given in [37].

In order to stabilize the circle fit across multiple data frames, a running average of 30 circles was implemented, and the mean circle was returned.

After fitting the circle, the estimated circle center and radius were used to scale the circle to a unit circle in order to stabilize the solution.

As discussed before, the extracted I/Q data lie on a circle in the complex plane [28], as the complex vectors rotate due to the radial velocity of the movement, increasing the phase [34]. In order to extract the phase containing the motion from this circle, several methods are used, the most commonly used being arc-tangent demodulation [4].

The displacement $\psi_{l}$ containing that motion is calculated as

(23) $$\psi_{l}=\arctan\left(\frac{Q(t)}{I(t)}\right).$$

By applying arctangent demodulation on the unit circle transformed I/Q data (Equation (23)), the phase could be obtained. It returned the phase angle in the interval [−π,π] [39]. After that, phase unwrapping was applied—it shifts the phase angles by adding multiples of 2π until the jump from one angle to its consecutive one is less than π, in order to restore the signal’s original phase values [40].

This extracted phase will contain more information than needed. As the goal was to extract only the pulsation curves, a 4th order Butterworth bandpass filter was applied, with cutoff frequencies of 0.75 Hz and 5 Hz. The upper bound was set to 5 Hz to minimize noise but still allowed the extraction of relevant morphology.

The returned signal will only contain frequencies between the specified cutoff frequencies.

In order to map the phase angle (in radians) to motion (in µm), the extracted displacement was multiplied by the transformation factor *t*. It was computed as

(24) $$t=\frac{\lambda /2}{2\pi \cdot 10^{6}},$$

where the wavelength λ was computed using Equation (4).

### 4.3. Feature Extraction

The extracted skin displacement will contain sequential pulse waves. For computing continuous beat-to-beat BP values, we thus needed to extract information for each individual pulse wave.

#### 4.3.1. Filtering

**Figure 7:**
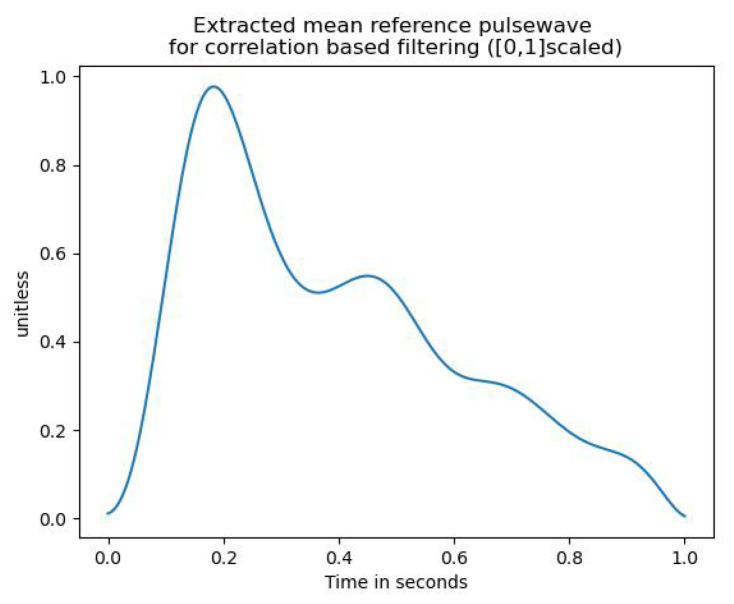
Extracted mean reference pulse wave for correlation-based filtering.

Before feature extraction, a label was assigned to each pulse wave, stating whether or not the given pulse wave followed the expected morphology (as e.g., in Figure 2) sufficiently well or is too noisy. This distinction was implemented using a correlation-based approach. In order to provide a quantitive measure of how well a given pulse curve resembles the expected morphology, the correlation between a reference wave that satisfies the expected morphology and the pulse curve in question was computed. This reference pulse wave was obtained as the mean pulse wave of five pulse waves from different subjects. This was performed by first interpolating all five curves to the same fixed length. Then, the curves were averaged. The resulting mean reference waveform is depicted in Figure 7.

The benefit of using a mean waveform is that it allows the filtering not to be solely based on the resemblance to a single subject. Thus, fewer pulse waves are discarded.

In order to make the reference pulse wave and the pulse wave in question comparable, both waves were [0, 1]-scaled to account for amplitude variability. The classification into usable and discardable pulse waves was then performed by applying thresholding over their Pearson correlation coefficient. That means, if the correlation was greater than the threshold, it was classified as usable and discarded otherwise.

#### 4.3.2. Calibration

As already mentioned, the radar measures skin displacement in µm as opposed to directly measuring pressure in mmHg units. It was, therefore, necessary to implement a calibration procedure to enable mapping from displacement to pressure [16].

The procedure must be calibrated at least once at the beginning of the measurement. However, it is beneficial to include multiple measurements when recording for longer periods [1,16]. Yet, some research suggests that this initial calibration is effective for longer periods than expected, even after one month [2]. Nevertheless, implementing calibration methods is crucial for meaningful BP estimates [41] and “is the key to improving the accuracy and robustness of non-invasive continuous blood pressure estimation” [1]. Its importance can thus not be stressed enough.

In order to calibrate, the pressure values of the brachial artery cuff were used [42].

The scaling factor for stretching the small amplitude skin displacement curves to the larger amplitude blood pressure curves from the reference device was computed as:

(25) $$\mathrm{scaling}\_\mathrm{factor}=\frac{\mathrm{refSYS}-\mathrm{refDIA}}{\max(\mathrm{first}\_\mathrm{good}\_\mathrm{PW})-\min(\mathrm{first}\_\mathrm{good}\_\mathrm{PW})}.$$

refSYS and refDIA were extracted from the reference device’s arm cuff values and used as the one-time calibration values. The ratio of the differences between the highest and lowest points of reference device and radar curves served as a scaling factor. The first good pulse wave acted as the reference radar pulse wave since using a pulse wave of unsatisfactory quality would distort the scaling factor.

Then, each individual pulse wave was scaled by applying:

(26) PW=(PW−min(PW))·scaling_factor+min(PW).

The first part of the equation, where the curve’s minimum was subtracted from the curve, shifted the pulse wave, so it then ranged from zero to the difference between its maximum and minimum value. That way, multiplying it with the scaling factor from Equation (25) yielded that the difference in amplitude was scaled to the appropriate range.

Adding the minimum of the pulse wave ensured that not all pulse waves began at zero, so information about the radar-extracted skin displacement amplitude was not entirely lost by the scaling process.

Additionally, the parameters of age, height, and weight were introduced as additional calibration features in [2,43]. The authors of [2] have shown that these parameters, additional to PPG-based waveform analysis, significantly reduced prediction errors. Therefore, all three of these parameters, as well as the subject’s gender, were included as features for the neural network in order further to stabilize BP mapping through this additional indirect calibration measure.

#### 4.3.3. Pulse Wave Analysis: Computing Features

Then, pulse wave analysis was performed on the calibrated, filtered wave.

The arterial pulse is considered to contain “an abundance of information on the health or disease of a patient” [5]. As discussed before, BP is one of the most dynamic physiological variables. Therefore, assessing these dynamic features of BP gives access to clinically relevant information—thus emphasizing the importance of waveform analysis. PWA is the morphological analysis of the pulse waveforms in order to extract information about cardiovascular health [5].

In this work, 21 features collected from the literature were extracted from the pulse waves. A combination of amplitude-based features and timing features was employed. The six amplitude-based features are enumerated in Table 1.

**Table 1:** Chosen amplitude-based features.

	Feature	Feature Description
1	Systolic blood pressure (SBP)	maximum value of the extracted pulse waveform [5]
2	Diastolic blood pressure (DBP)	minimum value of the extracted pulse waveform [5]
3	Pulse Pressure (PP)	PP=SBP−DBP [5]
4	Dicrotic Notch	trough between SBP and secondary upstroke
		due to pressure wave reflection [5]
5	Pressure at time of reflection (PR)	value at the beginning of the backward
		reflection of arterial pressure
		inflection point PR occurs after the SBP point
		in young subjects and before in old subjects
		due to increased arterial stiffness [10], see Figure 8
		different types of PR occurrences are visualized in Figure 9
6	Augmented pressure (AP)	describes the BP increase due to early
		arrival of the reflected wave, as visualized in Figure 8.
		$AP=\left(SBP-PR\right)\cdot AI_{x}$
		$AI_{x}=1$ marks arrival before systole
		and $AI_{x}=-1$ after.
		AP is thus positive for older subjects and
		negative for younger ones and
		indicates arterial stiffness due to age [5,44].
		Figure 9 shows four different types of PR
		and their resulting AP that increases with age
		as the reflection of the pressure wave occurs
		increasingly earlier (type B and A) before it
		eventually vanishes (type D).

The other features were timing-based, and are summarized in Table 2. For our chosen radar configuration, 250 samples comprised one second of data; we denote this number as sps.

To obtain the time in seconds we, therefore, divided the index of occurrence by sps. The duration of one cardiac cycle is determined by the length of the detected pulse wave, which in turn is highly related to the heart rate.

**Figure 10:**
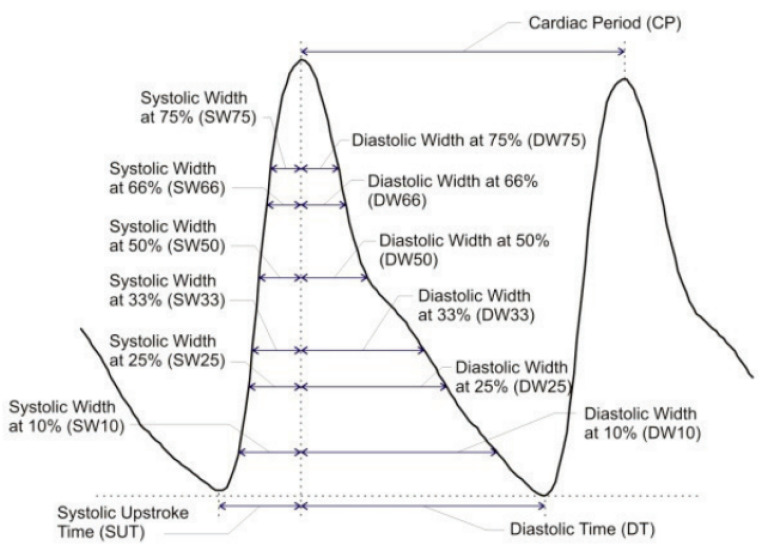
Sketch of systolic and diastolic widths at given pulse pressure level. Reprinted with permission from [6]. *©* 2013, IEEE.

In turn, the heart rate itself is highly related to BP values [45]—the shorter one cardiac cycle takes, the higher the BP becomes [6]. In order to capture as much timing information as possible, the authors of [6] suggested estimating the systolic and diastolic duration at various PP ranges. This is visualized in Figure 10. Since the estimation procedure is the same for all width-based timing features, it will be outlined once below instead of repeating the procedure for each feature.

First, the pulse wave height at the respective percentage of the PP was estimated as

(27) h=(DBP+PercentageOfPP).

Then, the intersection of this height with the pulse waveform was calculated by following the procedure from [46]. The intersection locations were computed by subtracting the pulse curve and height from each other and computing the location of sign changes of this difference—the first being the intersection with systole and the second the intersection with diastole. Therefore, the systolic width or systolic duration was calculated as the difference between the SBP index and the first intersection, divided by sps to obtain time duration. The diastolic width or diastolic duration was calculated as the difference between the second intersection and the SBP index, divided by sps.

After each pulse wave, the extracted features were appended to the database, together with the calibration features of age, gender, height, and weight. This database was then used to train neural networks for regression.

## 5. Experimental Setup

**Figure 11:**
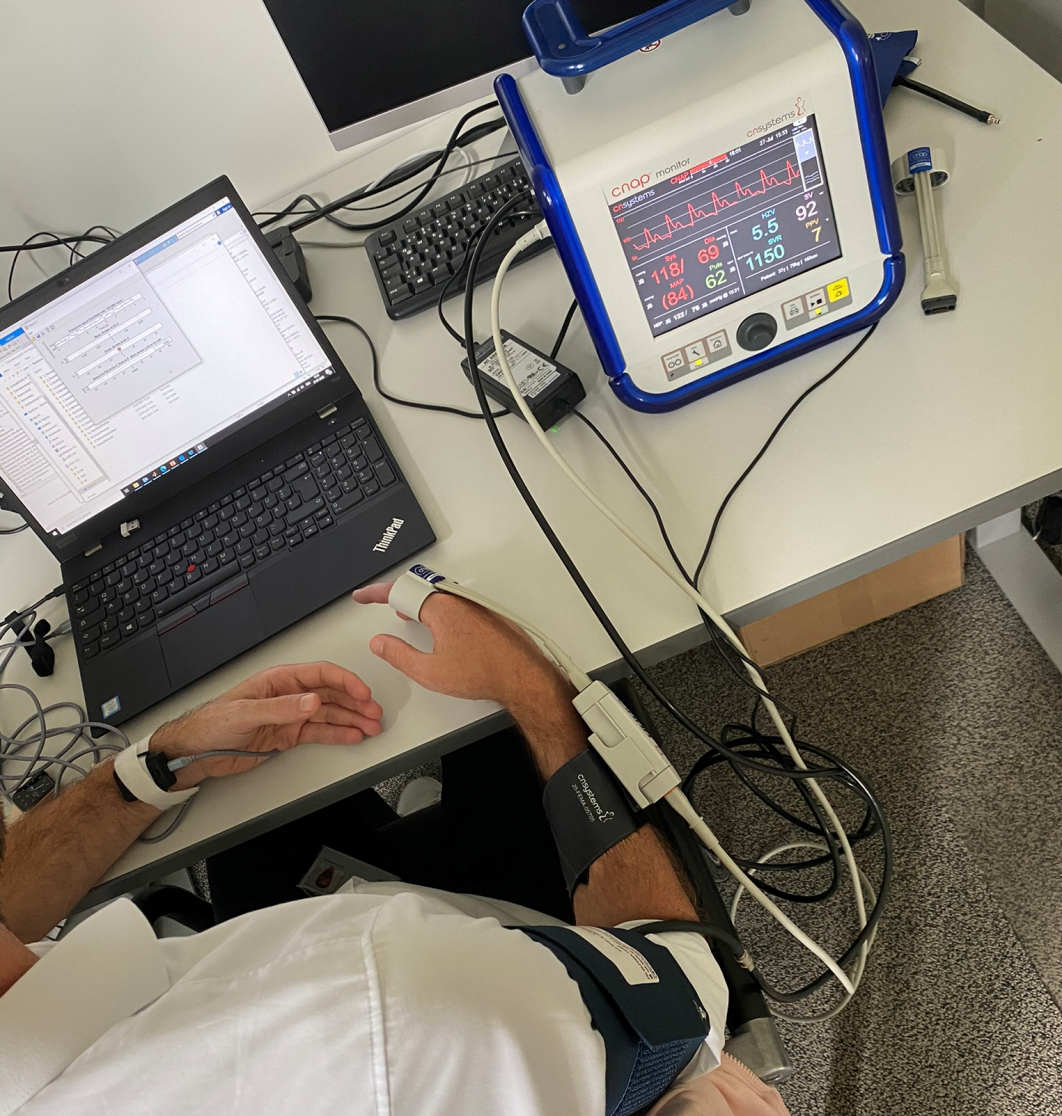
Data campaign setup.

This section covers the experimental setup for simultaneous data acquisition from a reference device and the radar, as depicted in Figure 11.

**Figure 12:**
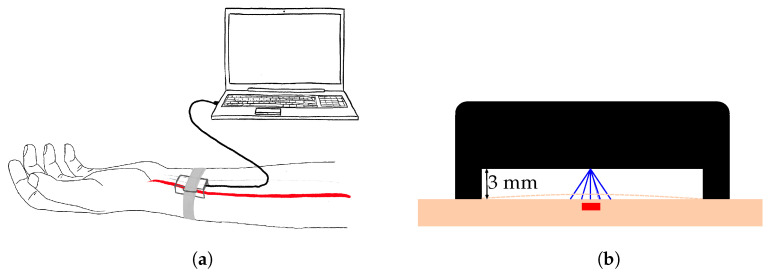
Visualization of the radar setup for data collection. (**a**) Radar positioning at the left wrist, above the radial artery. Radar is inside a 3D printed enclosure and streams the collected radar data to the PC via standard USB connection. (**b**) Close up view of the radar positioning. The red square represents the radial artery. The radar is located in the 3D enclosure (represented in black), with a standoff of 3mm. The blue lines represent the reflected electromagnetic waves from different directions.

The radar was held by a 3D-printed enclosure with a standoff of 3 mm and fixed to the left wrist with a velcro strap, as visible in Figure 12a. Special care was taken to position the radar above the radial artery, as depicted in Figure 12b. For that, the DOA, as determined by the method described in Section 4.1, was utilized. The radar was shifted until the DOA was in the previously mentioned expected range of [−10°,10°]. Only then was the data acquisition started, to ensure a higher quality of data and avoid the recording of noise only.

In order to keep distance of the radar from the skin, a 3 mm standoff wasadded to the enclosure. The goal was to extract the skin displacement caused by the vasomotion of the radial artery.

Section 5.1 describes the chosen radar and its configuration for the data collection campaign. Then, Section 5.2 describes the chosen reference BP monitoring device. In Section 5.3, the data collection is described. Finally, Section 5.4 provides insights into the collected data.

### 5.1. Radar Configuration

**Figure 13:**
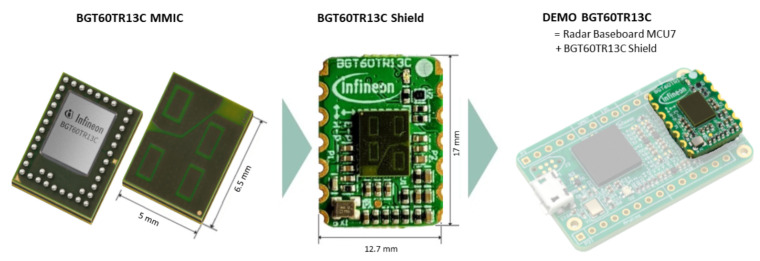
BGT60TR13C radar from Infineon Technologies AG. Source: [47].

The chosen radar for this work was the BGT60TR13C radar from Infineon Technologies AG (Munich, Germany) and is depicted in Figure 13. The BGT60TR13C is a 60 GHz radar sensor and has a form factor of 5 mm by 6.5 mm. For the data acquisition, the radar was placed on its shield and then on a Radar Baseboard MCU7. The PCB is equipped with a USB interface that allows data transmission to a host computer.

For integration of the radar into a wearable device, the baseboard would not be needed anymore. The compact size of the radar allows easier integration into wearables.

It has four integrated antennas, which are depicted as light green rectangles in Figure 13. One antenna is used for transmitting (upper left antenna in Figure 13) and three for receiving [48]. The first receiver antenna is the upper right, the second the lower left, and the third the one at the bottom right patch in Figure 13. The receiver antennas were placed in an L-shape (as visualized in Figure 13) such that both vertical and horizontal angles of arrival could be estimated.

Generally, the radar’s sensitivity in capturing minor motion increases with emitted frequency. This is because higher frequencies produce smaller wavelengths—thus, periodic motion comprises more wavelengths and therefore increases the signal’s phase [29].

Consequently, we chose the BGT60TR13C as it is a high-frequency 60 GHz radar that can be configured in compliance with different national radio emissions regulations, for example from ETS(Europe) or FCC(USA) [49].

The radar was flashed to the newest radar firmware *v.2.55*. For communication with the radar, the latest Radar System Development kit version *v.3.2.3* by Infineon Technologies was used, which can be employed in MATLAB^®^. The radar sensor was first connected to the computer with a USB cable to start the data acquisition.

The chosen radar settings were then used to configure the radar sensor: Since the range resolution is inversely proportional to the bandwidth (Equation (6)) and the radar was in closest proximity to the skin, the bandwidth was set to its maximum value. For the BGT60TR13C, the lower frequency was set to 58 GHz and the highest frequency to 63.5 GHz. Using Equations (3) and (6), a range resolution of roughly 2.7 cm was obtained. Targets could thus be spatially distinguished with these settings if they were at least 2.7 cm apart.

We utilized 32 samples per chirp and 256 chirps per frame were utilized, with a PRT of 0.004 s, providing a maximum range of 87.3 cm. Only the returned signal from the first non-DC range bin was utilized for skin displacement extraction; all information from greater distances was hence ignored. Given the fact that the PRT was 0.004 s, the number of samples per second was obtained as $\frac{1s}{PRT}=\frac{1s}{0.004s}=250$.

### 5.2. Reference Device

The CNAP^®^ device from CNSystems (Graz, Austria) provides continuous and accurate blood pressure readings, as well as the raw pulse wave data with timestamps. This allows for morphology comparisons between the reference device- and the radar-extracted pulse waves. The technology employed in this device is vascular unloading. After a one-time calibration, the arm cuff is no longer used. Instead, pressure is built up in the finger cuff.

**Figure 14:**
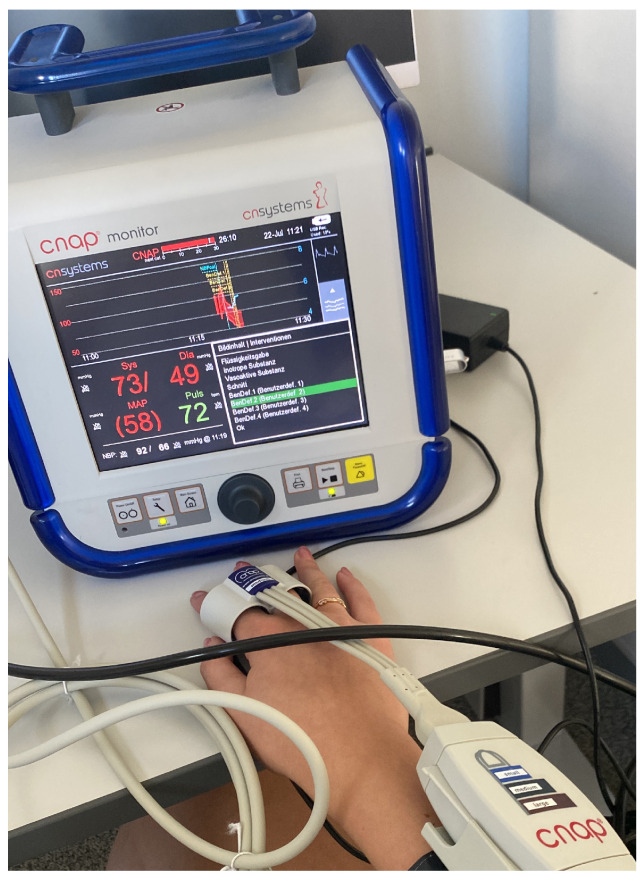
Close up of BP trend, estimated from CNAP^®^ device.

During the data campaign, tags were used in order to mark when the radar acquisition has started in order to be able to synchronize the pulse waves afterward. The bottom left window of the CNAP^®^ monitor (Figure 14) shows the systolic (Sys), diastolic (Dia), as well as the mean arterial blood pressure (MAP) readings for each pulse wave, and the current heart rate (Puls).

### 5.3. Data Collection

Before starting the data acquisition, the subject was asked not to drink coffee or eat for at least 15 min before recording as this would lead to increased blood pressure. After the subjects arrived, they were asked to sit without crossing their legs and to relax. Then, the subject was connected to the CNAP^®^ system and the radar system, as visualized in Figure 11.

The goal for the setup was to take at least five minutes [14] without letting the subjects know that they were made to sit for 5 min before starting the recording. This way, the blood pressure would have time to return to its regular state since walking to the data acquisition site also increases blood pressure.

For the CNAP^®^ system, firstly, the appropriate arm cuff and finger cuff sizes were determined and connected to the subject’s right side of the body and the CNAP^®^ data acquisition was started. Following that, the wrist-worn radar setup was connected to the left wrist, utilizing the estimated angle information to optimally place the radar. When starting the software, the subject was first asked for their consent for the data acquisition and anonymized collection of personal data such as age, gender, height, and weight. If consent was not given, the data acquisition was abandoned as these are vital parameters for calibrating the BP mappings, see Section 4.3.2.

The answers to the subject characteristics were entered by the author so that the subjects would move as little as possible and remain relaxed. For later time alignment, the radar acquisition was started simultaneously by pressing an intervention button at the CNAP^®^. Since the data acquisition for the CNAP^®^ device started earlier than for the radar, the set intervention was later used to synchronize the data. After 15 min, the data acquisition was stopped simultaneously.

### 5.4. Data Demographics

During the data acquisition, data from 55 subjects were used to build the feature database. Out of these 55 subjects, 12 were female and 43 were male (none identified as diverse), leading to an uneven distribution of 21.8% females and 78.2% males.

**Figure 15:**
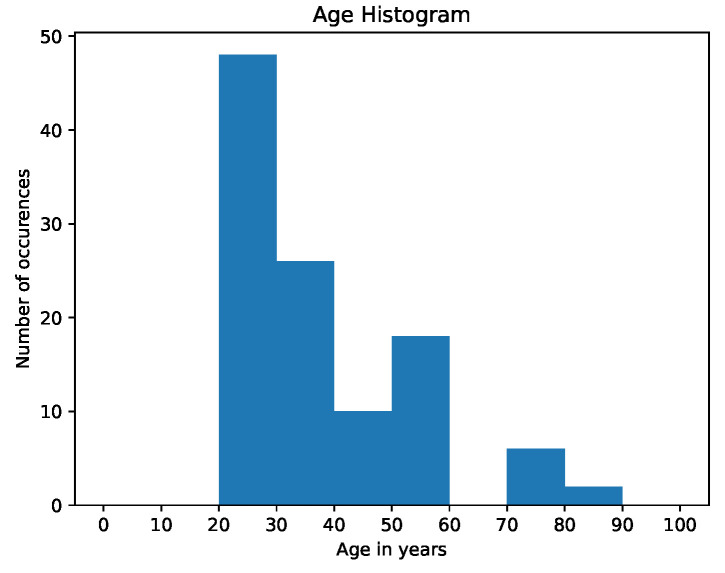
Clustered age histogram of study subjects.

This leads to an imbalanced dataset, which is biased towards males as most of the data were recorded within a technical working area. Therefore, male overrepresentation was expected. Figure 15 shows the distribution of subject age in decades—as visible, a wide range of age is represented. However, subjects in their twenties and thirties are over-represented.

This clearly leads to the dataset being biased toward the younger population. However, the dataset also contains elderly subjects above the age of 70.

Three databases were created for this study: one for each correlation threshold, i.e., 0.7,0.8,0.9. The only difference between these databases was the assigned usable/discardable label per pulse wave—the extracted features are the same for all thresholds.

The Association for the Advancement of Medical Instrumentation (AAMI) has certain requirements for the subject characteristics of the obtained dataset [50]. These are summarized in Table 3. We added our dataset characteristics to the table and highlighted in bold where we met the requirements. As Table 3 shows, the number of subjects is insufficient and there is a special need to add more females to the dataset to make it more balanced. While the data contained a sufficient ratio of lower blood pressure values, more hypertension subjects must be added for future research. However, this issue might be resolved by adding more subjects in general. For a first feasibility study, this dataset is still considered sufficiently balanced and will be enhanced in future research.

## 6. Results

The feature database was built by looping over all subjects and using their respective radar and CNAP^®^ data. For each subject, the whole radar data were divided into individual pulse waves. Sometimes, the waveforms appear to be inverted—the cause of this phenomenon remains a matter for future research. If the data were indeed inverted, the whole waveform series was multiplied by −1 in order to revert the inversion. The process of determining whether a waveform chain was inverted was performed manually. However, automating the detection of inversion will be implemented in the future.

This section first gives an overview over the algorithm’s skin displacement extraction results. It then outlines filtering results. Finally, the results from training neural networks for regression are presented.

### 6.1. Skin Displacement Extraction

**Figure 16:**
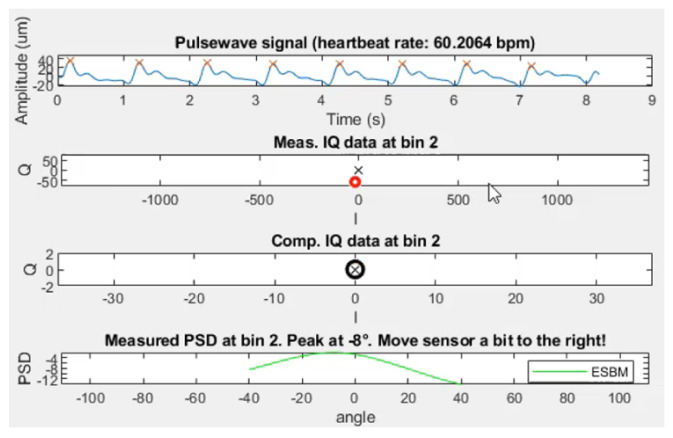
Result of live pulse wave extraction for subject 39.

The resulting skin displacement extraction attached to a subject’s wrist has proven to be capable of extracting the smallest movement, as small as a few µm. An example of live extraction of skin displacement of subject 39 can be seen in Figure 16.

The data acquisition worked exceptionally well for this subject, producing periodic pulse waves of roughly 50 µm amplitude. The algorithm captured the pressure morphology exceptionally well, being able to extract not only the systolic peak location but also the dicrotic notch and diastolic peak. The fact that the DOA was −8 ° proves the claim that a DOA in the range [−10 °;10 °] is sufficient.

**Figure 17:**
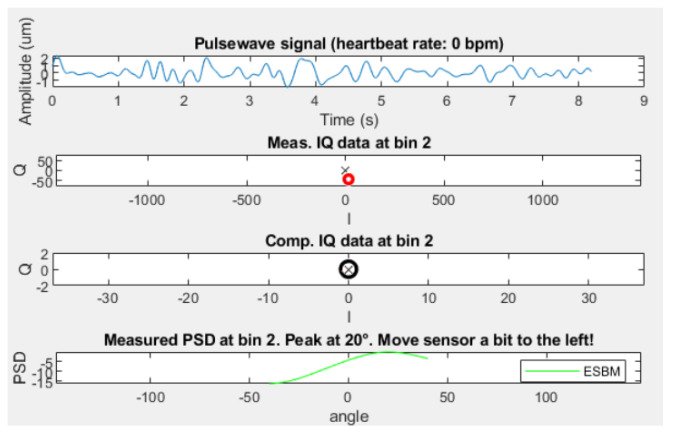
Result of live pulse wave extraction when the sensor is placed on the table and records only noise.

To demonstrate that the extracted waves are well above noise level, Figure 17 depicts an example where the radar device is placed on the table, antenna side up. Not only is the DOA at 20 °, but the pulse wave signal also shows random non-periodic movement in the range of maximum 3 µm.

**Figure 18:**
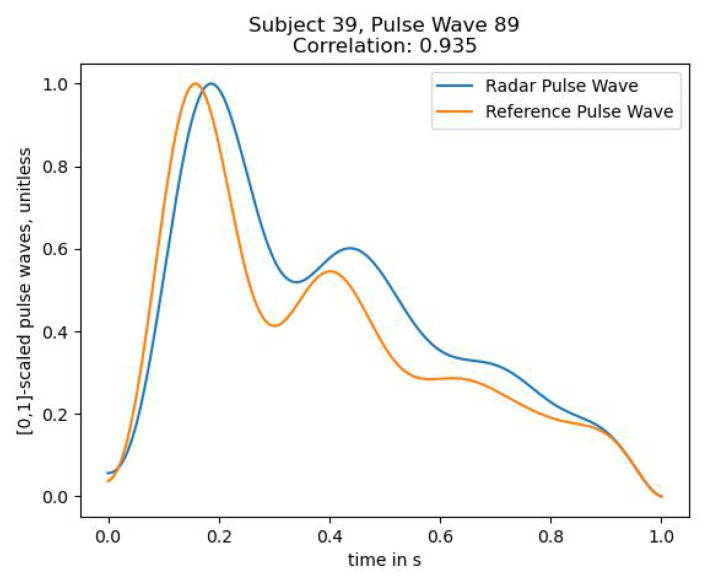
Comparison of [0, 1]-scaled pulse wave of radar and CNAP^®^. Correlation value shows the correlation between this wave and the reference pulse wave, not the CNAP^®^ curve.

Figure 18 shows a plot of the [0, 1]-scaled extracted pulse waves from both radar and the CNAP^®^ reference blood pressure device. The figure clearly displays that the skin displacement matches the expected wave morphology from the reference device, only the diastolic peak is slightly higher.

These figures demonstrate that the algorithm is very capable of extracting skin displacement that shows a meaningful resemblance to the expected pressure waveform morphology.

**Figure 19:**
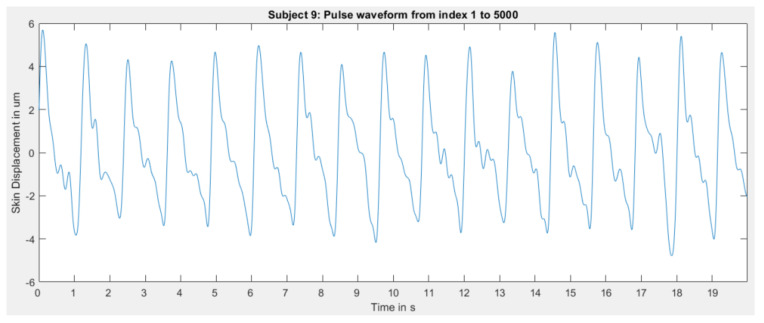
Extracted pulse waves for subject 9.

However, the algorithm did not perform equally well for each subject. For some subjects, skin displacement extraction worked remarkably well, as demonstrated above. However, some extractions were slightly noisy, for example for subject 9 in Figure 19.

**Figure 20:**
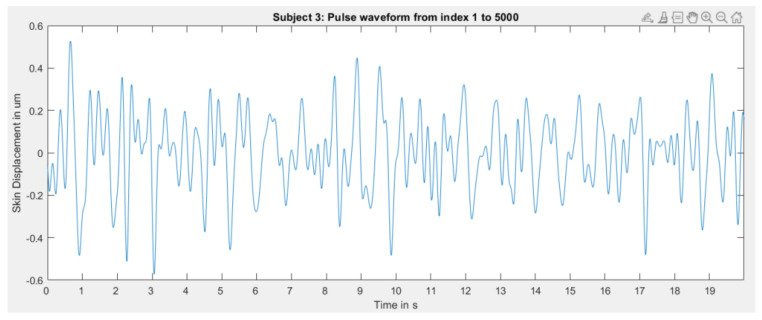
Extracted pulse waves for subject 3.

Some recordings produced such deficient results that it is likely that the radial artery was not located well enough since it seems that only noise was recorded, see Figure 20.

Not only is the amplitude of extracted skin displacement extremely low, but it also does not resemble the expected morphology.

Nonetheless, it must be noted that, for this subject specifically, manually locating the artery by using the index finger to locate the pulsation origin was also difficult.

It can thus be concluded that extracting the skin displacement using radar is indeed possible with the provided signal processing algorithm. Sometimes the extracted waves are slightly noisy—likely when the artery is not located well enough. Under some circumstances, extraction seems very difficult. The reasons for that difficulty are small arterial pulsations from the subject, i.e., very small skin displacement, not locating the radial artery well enough, and the distance from the radar to the skin. Since the sensor is even closer to the skin than the first range bin (here 2.7 cm), it is very likely that this has a tremendous impact on signal quality. Analyzing the reasons stated above remains a matter of future research.

### 6.2. Filtering Results

The filtering was performed three times, each time with a different correlation threshold.

Table 4 shows the percentage of usable pulse waves per correlation threshold. As expected, the amount decreases with higher correlation thresholds as a deviation from the reference pulse wave is restricted more.

### 6.3. Neural Network Training Results for Blood Pressure Value Regression

Neural networks are powerful tools that were utilized to train blood pressure mapping. In order to find a good neural network for predicting blood pressure using the extracted features, 126 fully connected feedforward networks were trained.

Various databases for different correlation thresholds were created. These were 0.7, 0.8, and 0.9. This was carried out in order to analyze whether it is more beneficial for learning to use more—but likely worse quality—data, or fewer data that fulfil higher quality standards. Essentially, this approach serves to analyze the bias-variance tradeoff where more data means more variance and fewer data mean less variance and potentially higher bias. The extracted features were not affected by this thresholding, only their assigned label. The database for training the neural network contained only those pulse waves that were classified as usable ones, i.e., those whose correlation exceeded the threshold.

The input features were preprocessed first by normalizing each feature column so that values of this feature lie in the range [0,1]. One reason for applying normalization is that the features vary in scale—bringing all features to the same range helps to stabilize the fitting procedure [51].

Subsequently, the total database was shuffled and split into a training set and a testing set, with 80% used for training and 20% used for testing. The random state was set to 42. The value assigned to the random state variable controls the splitting of data and ensures that the same split is obtained when repeatedly splitting the data.

**Figure 21:**
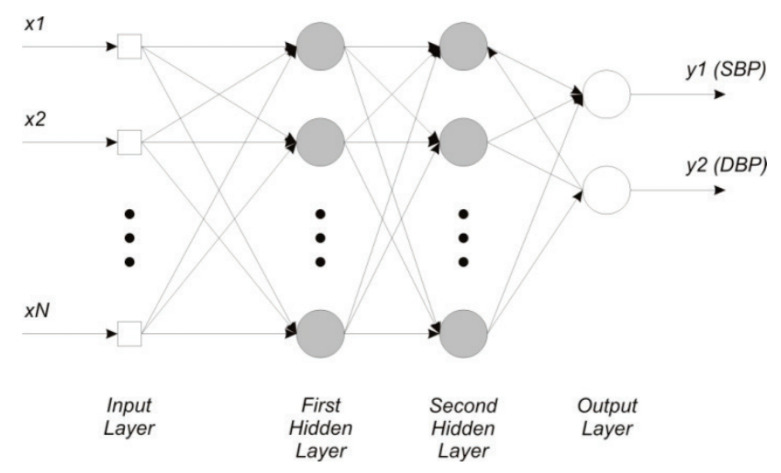
Sketch of a network used for blood pressure estimation. The figure shows the mapping from N features to SBP and DBP, using two hidden layers. Reprinted with permission from [6]. *©* 2013, IEEE.

Several network topologies were used for each of these thresholds to fit the input data to the expected output. Only two hidden layers were used, since this work presents a study of general feasibility. For further optimization, more hidden layers could be utilized. A general two hidden layer network topology is sketched in Figure 21.

The input layer has 25 input nodes, one for each feature. The first hidden layer had a varying size from 30 to 60 nodes, with an incremental increase of 5 nodes. The second hidden layer had either 5, 10, or 15 nodes, and the output layer had two nodes—one for the SBP prediction and one for DBP prediction. In order to decrease the weight of potentially unimportant features, L1 regularization can be applied to the input layer. Therefore, all network architectures were trained twice—once without regularization, and once with L1 regularization. In total, 42 networks have been trained per correlation threshold. The chosen activation function was the ReLU since it forces a positive output. The training process minimizes prediction error by iteratively adapting model parameters.

As regression is used to predict blood pressure pairs, the mean squared error of the predicted value compared to the ground truth was used as the loss function. The model parameters were optimized using the Adam optimizer, with a validation split of 15%. This means that 15% of the training data were withheld from the training process.

Instead, model performance is evaluated after each epoch by testing on the validation set.

Each network was trained for 50 epochs, with a batch size of 10. This means that the model iterates through the training set 50 times. During each epoch iteration, ten samples at a time will be fed into the network and used to update model parameters until the model has seen all samples of the training set.

The root mean squared error (RMSE) was chosen as a regression metric. Metrics are used to monitor the model’s performance during training. The errors are calculated as the difference between ground truth and prediction, and their mean error is calculated by averaging all of the squared errors. Finally, the predictive quality was tested by applying the trained model to the previously unseen testing data.

Training for different thresholds was performed in order to determine whether more data of worse quality or fewer data of better quality would produce better outputs. Increasing the correlation threshold leads to fewer data being used to train the networks. Hence, data variance is decreased and its bias is increased.

The AAMI has additional requirements for the validation of non-invasive blood pressure measuring devices [50]. These are summarized in Table 5.

The best network per correlation threshold is chosen by selecting the neural network that produces the smallest RMSE for that threshold. Table 6 gives an overview of the results of applying the best model per correlation threshold on its test set, where a bold number means that this error is within AAMI bounds for non-invasive blood pressure monitoring.

Additionally, the performance of our small networks was compared to the British Hypertension Society standard for BP measuring [52], which is summarized in Table 7.

We compared our minimal network models to the BHS standard in Table 8—for the chosen small model architectures, the results were insufficient compared to the BHS standard. In particular, the percentage requirement for errors below 5 mmHg were not reached. Here, the authors would like to point out that reaching this standard was not a priority of the presented work, but rather supplying the reader with an in-depth radar signal processing pipeline and a set of meaningful features. For a very shallow network and limited training time, this still shows a great feasibility of the approach. Deeper network architectures are very likely to improve these results.

For correlation threshold 0.7, the best neural network was “25-45-10-2” (thus, two hidden layers with 45 and 10 nodes, respectively) trained with L1 regularization, where the RMSE was 11.7 mmHg. This network returned 9.7±8.5 mmHg (mean ± standard deviation) error for systolic pressure values and 8.3±6.0 mmHg for diastolic pressure values.

**Figure 22:**
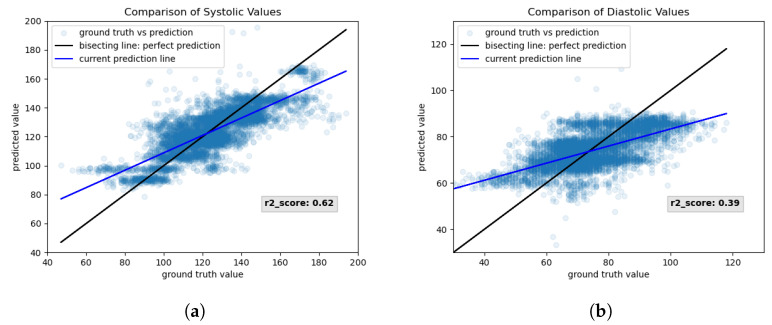
Results of training neural network with 25 input nodes, two hidden layers with 45 and 10 nodes, respectively, and 2 output nodes. Used correlation threshold for filtering: 0.7. Regularization: L1 regularization on input features. (**a**) Scatter plot of systolic values. (**b**) Scatter plot of diastolic values.

Figure 22 shows the results, loss, and metrics of the chosen network for correlation threshold 0.7. Figure 22a,b show the scatter plots of systolic and diastolic values, respectively.

For each pulse wave in the testing set, the ground truth value (x-axis) is plotted against its predicted value (y-axis). For a perfect mapping, the prediction lies on the bisecting line of the coordinate system. For easier visualization, a line—visualized in blue—is fitted through the scatter plot. That way, deviation from the perfect bisecting line is visualized more clearly. The output of the predicted diastolic values seems to be restricted to a certain range since values below 60 mmHg are always mapped to roughly 60 mmHg and do not follow the bisecting line. Similarly, all values above 90 mmHg seem to be clipped to 90 mmHg. The r2-score for systolic and diastolic predictions is indicated in Figure 22. A perfect model that captures data variability optimally will have an r2-score of 1. As visible from Figure 22, the r2-score for systolic values is higher than for diastolic values.

**Figure 23:**
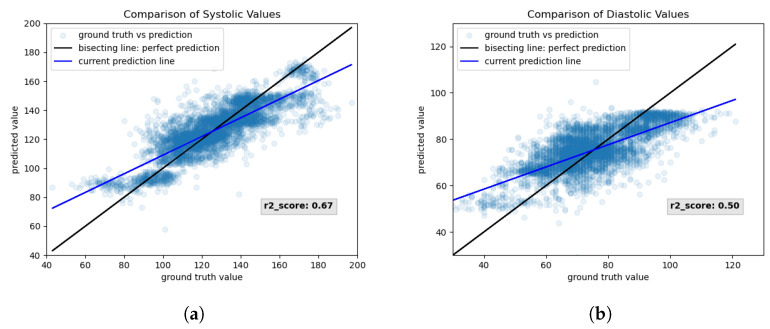
Results of training neural network with 25 input nodes, two hidden layers with 50 and 15 nodes, respectively, and 2 output nodes. Used correlation threshold for filtering: 0.8. Regularization: None. (**a**) Scatter plot of systolic values. (**b**) Scatter plot of diastolic values.

For correlation threshold 0.8, the best neural network was “25-50-15-2”, trained without regularization, where the RMSE was 11.0 mmHg. This network returned 9.2±8.3 mmHg (mean± standard deviation) error for systolic pressure values and 7.7±5.7 mmHg for diastolic pressure values. Figure 23 shows the training results that were estimated during the training of network “25-50-15-2” for correlation threshold 0.8. Again, the systolic (Figure 23a) and diastolic (Figure 23b) scatter plots show that they follow the bisecting line sufficiently well. The mapping of diastolic values is better than for the network in Figure 22 since the fitted scatter plot line is closer to the perfect bisecting line.

This claim is additionally backed by the fact that the r2-score is higher for both systolic and diastolic predictions.

For correlation threshold 0.9, the best neural networks were “25-45-15-2” and “25-60-10-2”, both trained without regularization and with an RMSE of 11.7 mmHg.

The former network returned 9.9±8.9 mmHg (mean± standard deviation) error for systolic pressure values and 7.9±5.9 for diastolic pressure values, and the latter 10.2±8.9 mmHg and 7.5±5.8 mmHg, respectively.

**Figure 24:**
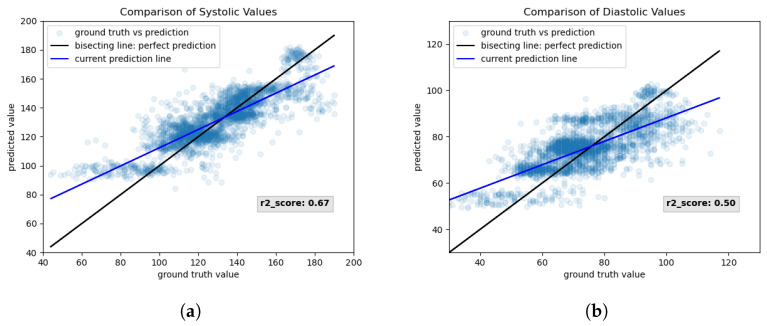
Results of training neural network with 25 input nodes, two hidden layers with 45 and 15 nodes, respectively, and 2 output nodes. Used correlation threshold for filtering: 0.9. Regularization: None. (**a**) Scatter plot of systolic values. (**b**) Scatter plot of diastolic values.

Figure 24 shows the results of applying the network “25-45-15-2” from correlation threshold 0.9 on the test set. Here, the scatter plots show that the systolic (Figure 24a) and diastolic (Figure 24b) predictions fall slightly better onto the bisecting line. The clipping of values in higher blood pressure values seems to have been resolved, while the r2-score remained the same as for correlation threshold 0.8. Only the prediction of lower values of blood pressure seems to surpass a lower threshold.

As discussed previously, the prediction of blood pressure values seems to be restricted to a certain output range. At first glance, this could look as though the network is producing bad predictions by requiring a minimum output or suppressing the output below a maximum. However, upon closer ground truth inspection it becomes apparent that the ground truth contains data that is in an unlikely range, e.g., a systolic BP reading of 40 mmHg.

Therefore, it can be argued that the reference device returned bad blood pressure estimates, e.g., when the finger cuff is not tight enough. Hence, the radar prediction might be even more favorable.

Next, contributing factors to successful blood pressure prediction are discussed. For that, the correlation of features with the ground truth systolic pressure and diastolic pressure are listed in Table 9 and Table 10, respectively. A higher correlation value of a feature with the ground truth value signifies a higher feature importance. The shown tables serve to visualize feature importance to the reader. This analysis was only performed for the database, which utilized 0.9 as the correlation threshold, since it showed the most promising results.

The reason for this assumption is that training neural networks on this database produced the most results where the mean systolic and diastolic errors were below 5 mmHg.

For the systolic pressure, height, age, gender, weight, and systolic upstroke time are the top five contributors to systolic blood pressure (see Table 9). All five of these show a positive correlation, i.e., the SBP increases when these features increase. This was an expected result since the four calibration parameters all relate to an increase in BP—e.g., it is well-known that BP increases with age. The features that least contribute to systolic BP prediction are SW25, SW10, DW33, DW75, and DW50.

For the diastolic pressure, height, pulse pressure, diastolic height, point of reflection, and systolic height are the top five contributors (see Table 10). Only the diastolic height shows a negative correlation—this is correct and expected since the pulse wave starts at a negative value due to the calibration procedure. The lower the diastolic point height is, the greater the DBP height is, in turn. The positive contribution of height was expected as well, as previously discussed. The fact that pulse pressure and systolic height are positively correlated to DBP prediction is likely explained by the following relation: systolic BP has a much wider range than diastolic BP. When the pulse pressure is large, it is hence likely that the systolic height increases.

It thus appears that higher systolic values allow for higher diastolic ones.

The features that least contribute to diastolic BP prediction are DW25, diastolic time, SW10, SW25, and DW33.

## 7. Discussion

A 60 GHz FMCW radar can extract the pressure waves with a remarkable resemblance to the ones from the reference device. However, despite being capable, pulse wave extraction produced unsatisfying results for some subjects. In fact, some results showed so little resemblance to expected waveform morphology that it seems that only noise was recorded. One reason might be that the underlying artery was not located precisely enough—this could be easily fixed by simply shifting the radar. Another reason could be that the artery’s expansion causes very little vasomotion and, thus, minimal skin displacement. When displacement is very small, a smaller arc is produced by the I/Q data, thus making circle fitting and skin displacement extraction more complex. This would lead to a bad signal-to-noise ratio and explain why some recordings seemed so noisy.

Thus, it can be stated that, while the radar suffers from limitations, it can extract pulse waves under the right circumstances. The problem of inverted waveform recordings will also be part of future research. A stability-analysis for the algorithm must be performed in order to determine how sensitive the radar is to blood pressure changes, and more and more balanced data must be collected on which the algorithm can be thoroughly tested and further optimized.

For the predictions, a clear correlation between ground truth and predicted values can be seen. While not reaching the AAMI, and BHS blood pressure measuring standards, the presented results were promising for a first feasibility study and will likely improve with further optimization and research. Still, our chosen approach shows great potential—all diastolic standard deviation errors satisfy the maximum error bound of 8 mmHg, being 6.0, 5.7, and 5.9 mmHg, respectively. The systolic standard deviation errors did not satisfy that criterium by being 8.5, 8.3, and 8.9, respectively. Still, they approach the requirement and therefore show great potential although the mean absolute error bound was not reached. Potential reasons for that are that models were only trained for very few number of epochs. It is possible that a model has reached a local, but not a global minimum during these short training periods. Additionally, using deeper network architectures is very likely to improve accuracy by being able to capture the feature relations to blood pressure better. Compared to the BHS standard, our trained models seem less promising. While at least 80% had errors below 15 mmHg, the models did not produce sufficient results of errors below 5 mmHg. Again, this issue is likely mitigated by using different and deeper architectures.

While we acknowledge that these results are insufficient for integration into a product, we would like to stress again that training an optimal model was not the core focus of the presented work. This work focuses on providing the reader with an in-depth radar signal processing pipeline and a set of meaningful features collected from the literature. We have shown that the extraction of pulse waves using radar is feasible and that even training with very shallow networks and the proposed features produced very encouraging results. Even in its current state, our approach would provide great value for at-home usage in the consumer market.

The RMSE per correlation threshold (Table 6) does not show significant variation. One explanation could be that the network models are very shallow. With deeper networks and longer training periods the RMSE would likely decrease even further and the benefit of using higher correlation, and thus higher data quality, would likely be enhanced. The RMSE of correlation threshold 0.8 seems to be even lower than for correlation threshold 0.9. One reason might be that the model for correlation threshold 0.9 reached a local error minimum rather than a global one. With longer training periods and deeper networks, the benefit of using higher-quality data would likely be enhanced and become more apparent. Training an optimal model, however, was not the core contribution of the presented work. Another reason for the better performance of the model for correlation threshold 0.8 is that it can capture blood pressure variability better by having more data with higher variance to train on.

This work has shown that features of extracted pulse waves contain valuable information for blood pressure mapping since using very shallow networks and few training epochs produced promising results. However, how stable this method is has to be analyzed, along with how often re-calibration is needed, and how sensitive the radar is to blood pressure changes. Additionally, this work provides a proof of concept only—different, and potentially deeper, architectures are likely to further improve prediction results.

In comparison to existing work, this approach utilized a PWA approach on a high-frequency 60 GHz FMCW radar.

The authors of [20] utilized a low-frequency 900 MHz CW radar and a PTT approach. This approach lacks ease of use since it requires the constant use of an ECG attached to the chest and of a PPG sensor attached to the earlobe. In contrast, our proposed approach requires only a single sensing modality that could be easily integrated into a wearable device. Using a higher frequency FMCW radar also allows for a better skin displacement extraction since the wavelength is smaller, and the frequency modulation additionally offers the advantage of range resolution.

While [22] presented a 24 GHz solution that showed promising results from 15 cm distance, it requires the subject to be sitting completely still in front of the radar. This limits the integration of this monitoring technique into daily life tremendously.

Our proposed approach does not suffer from this constraint as it is designed to be incorporated into wearable devices. Additionally, the use of a higher-frequency 60 GHz radar increases the radar’s sensitivity to minor motion extraction, as previously described.

Finally, the authors of [21] also utilized a 60 GHz FMCW radar sensor. However, while they did demonstrate that they could extract meaningful skin displacement, their work did not provide detailed insights into the required algorithmic steps. Additionally, this work did not present a feature selection set or an approach for mapping to blood pressure.

While the authors of [19] achieved great results with their two-channel ballistocardiogram solution, the significance of their research is limited by the small amount of data they acquired. Since only non-hypertensive subjects were studied, and only five blood pressure pairs per minute have been extracted, training of a regression network is easier as the expected output range is smaller. Additionally, their work was based on extracting heartbeat signals and not pulse waves—however, pulse waves are rich in information about the total cardiovascular state of the subject. Including pulse wave features is hence beneficial for predicting blood pressure. An advantage of our proposed approach is thus the extraction of blood pressure pulse waves. Finally, their proposed approach requires sitting down on the sofa, thereby limiting seamless integration into daily life. In contrast, our approach would allow integration into wearable devices after accuracy improvements.

## 8. Conclusions

There is a strong need for continuous, non-invasive, and cuffless blood pressure monitoring. A radar-based pulse wave extraction algorithm was presented that allowed pulse wave analysis on the individual pulse waves. After filtering and calibration, features of the extracted pulse curves of 55 subjects were used as input for training a neural network for regression, with their respective systolic and diastolic blood pressure pairs as target values. Networks for three different correlation thresholds were trained. The higher the correlation threshold, the higher the required similarity to a reference pulse wave is. Therefore, the quality of the passed pulse waves is higher and the network input will show less variance.

While the RMSEs for the best models per correlation threshold did not vary significantly, the network for correlation threshold 0.7 performed slightly worse than the ones for threshold 0.8 and 0.9. This is visible in the scatter plots for the diastolic values, where predicted values are clipped to a certain prediction range. The best network in terms of RMSE and mean and standard deviation errors was for correlation threshold 0.8 and not 0.9.

As discussed above, either the network for 0.9 did reach a local minimum instead of global one or the network benefits from a higher data variance.

In conclusion, using the aforementioned 25 features as input to a neural network-based regression produces very promising results. None of the 126 trained networks complied with the AAMI blood pressure measuring standard that requires a mean error of 5 mmHg and a maximum standard deviation of 8 mmHg, nor with the BHS standard.

However, the produced results showed great potential for a small network topology as a first feasibility study. By using larger networks and training using more epochs, this error could be potentially further decreased. Given the small network size, we conclude that the proposed features contain meaningful information that allow good predictive power. Hence, this approach will be utilized and refined for a potential industrial application. Incorporating this technique into wearable devices could provide continuous blood pressure readings across long periods of time, providing great insights into the patient’s cardiovascular system. Additionally, a wearable and cuffless device would increase patient comfort and therefore lead to more realistic blood pressure readings that are not distorted due to discomfort. It can therefore be concluded that radar-based blood pressure monitoring is indeed feasible and a promising approach that could be integrated into wearable devices for at-home blood pressure monitoring and screening applications.

## Figures and Tables

**Figure 8:**
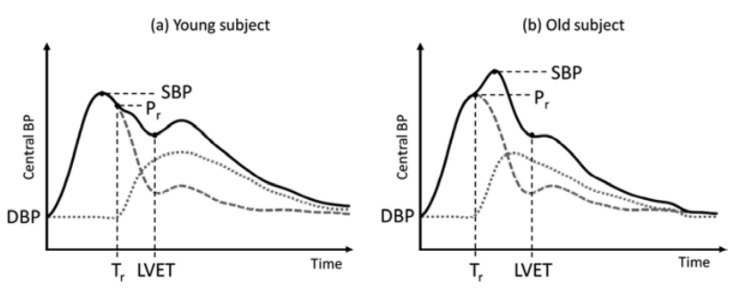
Visualization of difference in pressure curve morphologies for young and old subjects. Reprinted with permission from [5]. *©* 2019, Springer Nature Switzerland AG.

**Figure 9:**
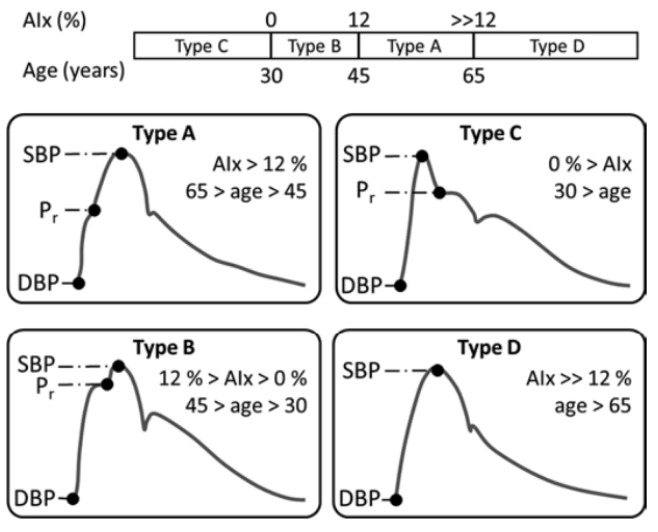
Visualization of typical blood pressure morphologies dependent on age group. Reprinted with permission from [5]. *©* 2019, Springer Nature Switzerland AG.

**Table 2:** Chosen timing-based features.

	Feature	Feature Description
7	Travel time of the reflected wave (TR)	describes the timing difference between the forward
		arterial pressure wave and its point of reflection
		TR=PR_index/sps
8	Systolic Upstroke Time (SUT)	describes the duration for the pressure wave to reach its peak
		SUT=SBP_index/sps
9	Diastolic Time (DT)	describes the remaining pulse wave duration after the SUT
		DT=(length(pulse_wave)−SBP_index)/sps
10–15	Systolic Width at *x*% of PP (SWx)	chosen pulse pressure levels: 10%, 25%, 33%, 50%, 66%, 75%
16–21	Diastolic Width at *x*% of PP (DWx)	chosen pulse pressure levels: 10%, 25%, 33%, 50%, 66%, 75%

**Table 3:** Data requirements in AAMI standard [50].

	AAMI	Our Obtained Data Set
number of subjects	>85	55
minimum percentage of females/males	>30%/>30%	21.8%/**78.2%**
systolic BP readings ≤100 mmHg	≥5%	**13.6%**
systolic BP readings ≥140 mmHg	≥20%	18.0%
systolic BP readings ≥160 mmHg	≥5%	3.7%
reference diastolic BP readings ≤60 mmHg	≥5%	**11.6%**
reference diastolic BP readings ≥100 mmHg	≥5%	**22.3%**
reference diastolic BP readings ≥85 mmHg	≥20%	3.0%

**Table 4:** Percentage of usable pulse waves per correlation threshold. Only pulse waves whose correlation with a reference pulse wave exceeds the threshold are considered usable.

Correlation Threshold 0.7	Correlation Threshold 0.8	Correlation Threshold 0.9
59%	45.5%	26.4%

**Table 5:** Performance requirements in AAMI standard.

AAMI	Mean Average Error SBP/DBP (mmHg)	Standard Deviation SBP/DBP (mmHg)
errors	≤5.0	≤8.0

**Table 6:** Results of applying the best trained model per correlation threshold on the test set.

Results	Correlation Threshold: 0.7	Correlation Threshold: 0.8	Correlation Threshold: 0.9
RMSE	11.7 mmHg	11.0 mmHg	11.7 mmHg
systolic error	9.7±8.5 mmHg	9.2±8.3 mmHg	9.9±8.9 mmHg
diastolic error	8.3±6.0 mmHg	7.7±5.7 mmHg	7.9±5.9 mmHg

**Table 7:** Characterization of BP measuring devices based on their cumulative error percentage.

	Cumulative Error (%)			
BHS		≤5 mmHg	≤10 mmHg	≤15 mmHg
	Grade A	60%	85%	95%
	Grade B	50%	75%	90%
	Grade C	40%	65%	85%
	Grade D	worse than Grade C		

**Table 8:** Comparison of our small network performances with BHS standard.

	Cumulative Error (%)				BHS Grade
		≤5 mmHg	≤10 mmHg	≤15 mmHg	
correlation threshold 0.7	SBP	35%	62%	80%	D
	DBP	34%	66%	86%	D
correlation threshold 0.8	SBP	37%	66%	82%	D
	DBP	40%	70%	89%	C
correlation threshold 0.9	SBP	33%	63%	80%	D
	DBP	37%	68%	89%	D

**Table 9:** Feature correlation for SBP prediction.

Feature	Correlation with SBP
Height	0.308105
Age	0.266924
Gender	0.255418
Weight	0.193005
Systolic Upstroke Time	0.191514
PP	0.178211
DBP	−0.175472
TR	0.162303
Diastolic Time	0.155195
DW10	0.151103
PR	0.129826
SBP	0.125036
AP	0.120960
SW66	0.110205
DW66	0.110205
SW50	0.105159
Dicrotic Notch	0.104861
SW75	0.101514
SW33	0.089672
DW25	0.082238
SW25	0.073401
SW10	0.070256
DW33	0.065650
DW75	0.053785
DW50	0.052848

**Table 10:** Feature correlation for DBP prediction.

Feature	Correlation with DBP
Height	0.283254
PP	0.160146
DBP	−0.145559
PR	0.119288
SBP	0.115125
AP	0.108361
Age	0.104587
Dicrotic Notch	0.090557
Gender	0.076756
TR	0.059479
Weight	0.053821
DW75	0.041253
Systolic Upstroke Time	0.024364
SW66	0.020781
DW66	0.020781
SW75	0.015191
SW50	0.015099
SW33	0.012003
DW10	0.010730
DW50	−0.008910
DW25	0.008178
Diastolic Time	0.006015
SW10	0.001647
SW25	−0.000496
DW33	0.000047

## Data Availability

The data are not publicly available due to internal company board policy.

## Abbreviations

The following abbreviations are used in this manuscript:

AO	Aortic valve
AP	Augmented pressure
BP	Blood pressure
CW	Continuous wave
DBP	Diastolic blood pressure
DOA	Direction of arrival
DT	Diastolic time
DW	Diastolic width
ECG	Electrocardiograph
ESBM-basedan an	Eigenspace based method
FFT	Fast Fourier Transform
FMCW	Frequency-modulated continuous wave
I/Q	In-phase/quadrature baseband signals
IVC	Inferior vena cava
LA	Left atrium
LV	Left ventricle
M	Mitral valve
MVDR	Minimum-variance distortionless response
PA	Pulmonary artery
PAT	Pulse arrival time
PP	Pulse pressure
PPG	Photoplethysmograph
PR	Pressure at time of reflection
PRI/PRT	Pulse repitition interval/time
PSD	Power spectral density
PTT	Pulse transit time
PWA	Pulse wave analysis
RA	Right atrium
ReLU	Rectified linear unit
RMSE	Root mean square error
RV	Right ventricle
SBP	Systolic blood pressure
SUT	Systolic upstroke time
SVC	Superior vena cava
SW	Systolic width
T	Tricuspid valve
TR	Traveltime of reflected wave

## References

[1] Barvik D., Cerny M., Penhaker M., Noury N. (2021). Noninvasive Continuous Blood Pressure Estimation from Pulse Transit Time: A review of the calibration models. IEEE Rev. Biomed. Eng..

[2] Xing X., Ma Z., Zhang M., Zhou Y., Dong W., Song M. (2019). An unobtrusive and calibration-free blood pressure estimation method using photoplethysmography and biometrics. Sci. Rep..

[3] World Health Organization Hypertension. https://www.who.int/news-room/fact-sheets/detail/hypertension.

[4] Buxi D., Redouté J.M., Yuce M.R. (2016). Blood pressure estimation using pulse transit time from bioimpedance and continuous wave radar. IEEE Trans. Biomed. Eng..

[5] Solà J., Delgado-Gonzalo R. (2019). The Handbook of Cuffless Blood Pressure Monitoring.

[6] Kurylyak Y., Lamonaca F., Grimaldi D. A Neural Network-based method for continuous blood pressure estimation from a PPG signal. Proceedings of the 2013 IEEE International Instrumentation and Measurement Technology Conference (I2MTC).

[7] Solberg L.E. (2016). Radar Based Central Blood Pressure Estimation. Ph.D. Thesis.

[8] Şentürk Ü., Yücedağ İ., Polat K. Repetitive neural network (RNN) based blood pressure estimation using PPG and ECG signals. Proceedings of the 2018 2Nd International Symposium on Multidisciplinary Studies and Innovative Technologies (ISMSIT).

[9] Fung Y. (1998). Biomechanics: Motion, Flow, Stress, and Growth.

[10] Davies J.I., Struthers A.D. (2003). Pulse wave analysis and pulse wave velocity: A critical review of their strengths and weaknesses. J. Hypertens..

[11] American Heart Association Understanding Blood Pressure Readings. https://www.heart.org/en/health-topics/high-blood-pressure/understanding-blood-pressure-readings.

[12] Nelson M.R., Stepanek J., Cevette M., Covalciuc M., Hurst R.T., Tajik A.J. (2010). Noninvasive measurement of central vascular pressures with arterial tonometry: Clinical revival of the pulse pressure waveform?. Proceedings of the Mayo Clinic Proceedings.

[13] Shaltis P., Reisner A., Asada H. Calibration of the photoplethysmogram to arterial blood pressure: Capabilities and limitations for continuous pressure monitoring. Proceedings of the 2005 IEEE Engineering in Medicine and Biology 27th Annual Conference.

[14] Yoon Y., Cho J.H., Yoon G. (2009). Non-constrained blood pressure monitoring using ECG and PPG for personal healthcare. J. Med. Syst..

[15] Johnson A.E., Pollard T.J., Shen L., Lehman L.w.H., Feng M., Ghassemi M., Moody B., Szolovits P., Anthony Celi L., Mark R.G. (2016). MIMIC-III, a freely accessible critical care database. Sci. Data.

[16] Proença M., Bonnier G., Ferrario D., Verjus C., Lemay M. PPG-based blood pressure monitoring by pulse wave analysis: Calibration parameters are stable for three months. Proceedings of the 2019 41st Annual International Conference of the IEEE Engineering in Medicine and Biology Society (EMBC).

[17] Fallow B.A., Tarumi T., Tanaka H. (2013). Influence of skin type and wavelength on light wave reflectance. J. Clin. Monit. Comput..

[18] Djeldjli D., Bousefsaf F., Maaoui C., Bereksi-Reguig F., Pruski A. (2021). Remote estimation of pulse wave features related to arterial stiffness and blood pressure using a camera. Biomed. Signal Process. Control.

[19] Seok W., Lee K.J., Cho D., Roh J., Kim S. (2021). Blood pressure monitoring system using a two-channel ballistocardiogram and convolutional neural networks. Sensors.

[20] Pour Ebrahim M., Heydari F., Wu T., Walker K., Joe K., Redoute J.M., Yuce M.R. (2019). Blood pressure estimation using on-body continuous wave radar and photoplethysmogram in various posture and exercise conditions. Sci. Rep..

[21] Johnson J.E., Shay O., Kim C., Liao C. (2019). Wearable millimeter-wave device for contactless measurement of arterial pulses. IEEE Trans. Biomed. Circuits Syst..

[22] Shi H., Pan J., Zheng Z., Wang B., Shen C., Guo Y. Radar-based blood pressure estimation using multiple features. Proceedings of the 2022 IEEE MTT-S International Microwave Biomedical Conference (IMBioC).

[23] Ludloff A. (1993). Handbuch Radar und Radarsignalverarbeitung.

[24] Winner H., Hakuli S., Wolf G. (2009). Handbuch Fahrerassistenzsysteme: Grundlagen, Komponenten und Systeme für aktive Sicherheit und Komfort: Mit 550 Abbildungen und 45 Tabellen.

[25] Infineon Technologies AG RADAR basics (FMCW). https://www.infineon.com/cms/media/eLearning/PSS/PSS_eLearning_1861_RADAR_basics_english/.

[26] Han K., Hong S. (2020). Phase-extraction method with multiple frequencies of FMCW radar for human body motion tracking. IEEE Microw. Wirel. Components Lett..

[27] Brooker G.M. Understanding millimetre wave FMCW radars. Proceedings of the 1st International Conference on Sensing Technology.

[28] Wang Y., Wang W., Zhou M., Ren A., Tian Z. (2020). Remote monitoring of human vital signs based on 77-GHz mm-wave FMCW radar. Sensors.

[29] Ahmad A., Roh J.C., Wang D., Dubey A. Vital signs monitoring of multiple people using a FMCW millimeter-wave sensor. Proceedings of the 2018 IEEE Radar Conference (RadarConf18).

[30] Zhao H., Gu X., Hong H., Li Y., Zhu X., Li C. Non-contact beat-to-beat blood pressure measurement using continuous wave Doppler radar. Proceedings of the 2018 IEEE/MTT-S International Microwave Symposium-IMS.

[31] Meigas K., Kattai R., Lass J. Continuous blood pressure monitoring using pulse wave delay. Proceedings of the 2001 Conference Proceedings of the 23rd Annual International Conference of the IEEE Engineering in Medicine and Biology Society.

[32] Feldman D.D., Griffiths L.J. (1994). A projection approach for robust adaptive beamforming. IEEE Trans. Signal Process..

[33] Capon J. (1969). High-resolution frequency-wavenumber spectrum analysis. Proc. IEEE.

[34] Hyun E., Jin Y.S., Lee J.H. (2016). A pedestrian detection scheme using a coherent phase difference method based on 2D range-Doppler FMCW radar. Sensors.

[35] Du N., Liu K., Ge L., Zhang J. ApneaRadar: A 24GHz radar-based contactless sleep apnea detection system. Proceedings of the 2017 2nd International Conference on Frontiers of Sensors Technologies (ICFST).

[36] Gouveia C., Albuquerque D., Vieira J., Pinho P. (2021). Dynamic digital signal processing algorithm for vital signs extraction in continuous-wave radars. Remote Sens..

[37] Taubin G. (1991). Estimation of planar curves, surfaces, and nonplanar space curves defined by implicit equations with applications to edge and range image segmentation. IEEE Trans. Pattern Anal. Mach. Intell..

[38] Al-Sharadqah A., Chernov N. (2009). Error analysis for circle fitting algorithms. Electron. J. Stat..

[39] MathWorks® Angle. https://de.mathworks.com/help/matlab/ref/angle.html.

[40] MathWorks® Unwrap. https://de.mathworks.com/help/matlab/ref/unwrap.html.

[41] Negishi K., Yang H., Wang Y., Nolan M.T., Negishi T., Pathan F., Marwick T.H., Sharman J.E. (2016). Importance of calibration method in central blood pressure for cardiac structural abnormalities. Am. J. Hypertens..

[42] O’Rourke M.F., Gallagher D.E. (1996). Pulse wave analysis. J. Hypertens. Suppl. Off. J. Int. Soc. Hypertens..

[43] Kachuee M., Kiani M.M., Mohammadzade H., Shabany M. Cuff-less high-accuracy calibration-free blood pressure estimation using pulse transit time. Proceedings of the 2015 IEEE International Symposium on Circuits and Systems (ISCAS).

[44] Avolio A.P., Butlin M., Walsh A. (2009). Arterial blood pressure measurement and pulse wave analysis—Their role in enhancing cardiovascular assessment. Physiol. Meas..

[45] Cattivelli F.S., Garudadri H. Noninvasive cuffless estimation of blood pressure from pulse arrival time and heart rate with adaptive calibration. Proceedings of the 2009 Sixth International Workshop on Wearable and Implantable Body Sensor Networks.

[46] von Rohr M. Intersection of Two Graphs in Python, Find the x Value. https://stackoverflow.com/questions/28766692/intersection-of-two-graphs-in-python-find-the-x-value.

[47] Infineon Technologies AG DEMO BGT60TR13C. https://www.infineon.com/cms/en/product/evaluation-boards/demo-bgt60tr13c/.

[48] Infineon Technologies AG BGT60TR13C. https://www.infineon.com/cms/en/product/sensor/radar-sensors/radar-sensors-for-iot/60ghz-radar/bgt60tr13c/.

[49] Infineon Technologies AG (2018). Health Effects of mmWave Radiation. https://www.infineon.com/dgdl/Infineon-Health%20Effects%20of%20mmWave%20Radiation-PI-v01_01-EN.pdf?fileId=5546d46266a498f50166f1ada0520444.

[50] Stergiou G.S., Alpert B., Mieke S., Asmar R., Atkins N., Eckert S., Frick G., Friedman B., Graßl T., Ichikawa T. (2018). A universal standard for the validation of blood pressure measuring devices: Association for the Advancement of Medical Instrumentation/European Society of Hypertension/International Organization for Standardization (AAMI/ESH/ISO) Collaboration Statement. Hypertension.

[51] Ming Chng Z. Using Normalization Layers to Improve Deep Learning Models. https://machinelearningmastery.com/using-normalization-layers-to-improve-deep-learning-models/.

[52] O’brien E., Waeber B., Parati G., Staessen J., Myers M.G. (2001). Blood pressure measuring devices: Recommendations of the European Society of Hypertension. BMJ.

